# Decoding VZV’s evolutionary arsenal: how Beijing strains use recombination and adaptive mutations to thrive

**DOI:** 10.1093/ve/veaf076

**Published:** 2025-09-23

**Authors:** Xiaotian Han, Changcheng Wu, Yao Deng, Lingfang Zhang, Lantao Wang, Zhongxian Zhang, Xuejie Zhang, Chen Mai, Wenjie Tan, Yang Zhao

**Affiliations:** Department of Pathogenic Biology, Hebei Medical University, Shijiazhuang 050017, China; National Key Laboratory of Intelligent Tracking and Forecasting for Infectious Diseases, National Institute for Viral Disease Control and Prevention, Chinese Center for Disease Control and Prevention, Beijing 102206, China; The Fourth Hospital of Hebei Medical University, Shijiazhuang 050010, China; National Key Laboratory of Intelligent Tracking and Forecasting for Infectious Diseases, National Institute for Viral Disease Control and Prevention, Chinese Center for Disease Control and Prevention, Beijing 102206, China; National Key Laboratory of Intelligent Tracking and Forecasting for Infectious Diseases, National Institute for Viral Disease Control and Prevention, Chinese Center for Disease Control and Prevention, Beijing 102206, China; National Key Laboratory of Intelligent Tracking and Forecasting for Infectious Diseases, National Institute for Viral Disease Control and Prevention, Chinese Center for Disease Control and Prevention, Beijing 102206, China; The Fourth Hospital of Hebei Medical University, Shijiazhuang 050010, China; National Key Laboratory of Intelligent Tracking and Forecasting for Infectious Diseases, National Institute for Viral Disease Control and Prevention, Chinese Center for Disease Control and Prevention, Beijing 102206, China; National Key Laboratory of Intelligent Tracking and Forecasting for Infectious Diseases, National Institute for Viral Disease Control and Prevention, Chinese Center for Disease Control and Prevention, Beijing 102206, China; National Key Laboratory of Intelligent Tracking and Forecasting for Infectious Diseases, National Institute for Viral Disease Control and Prevention, Chinese Center for Disease Control and Prevention, Beijing 102206, China; Department of Pathogenic Biology, Hebei Medical University, Shijiazhuang 050017, China; National Key Laboratory of Intelligent Tracking and Forecasting for Infectious Diseases, National Institute for Viral Disease Control and Prevention, Chinese Center for Disease Control and Prevention, Beijing 102206, China; Beijing You'an Hospital, Capital Medical University, Beijing 100069, China; Beijing Di'tan Hospital, Capital Medical University, Beijing 100015, China

**Keywords:** varicella-zoster virus, clade 2b.4, recombination, whole-genome sequencing, adaptive mutations

## Abstract

Varicella-zoster virus (VZV), a highly contagious α-herpesvirus, causes chickenpox and shingles. Although vaccines have been widely deployed, breakthrough infections still occur occasionally. Therefore, genomic surveillance of VZV remains essential. This study collected samples from 28 VZV-infected patients in Beijing, generating 25 complete viral genome sequences. These strains exhibited high genomic similarity and all belonged to Clade 2, which we further subdivided into five subclades with distinct characteristic variants. Most newly sequenced strains carried the A20795T (gC: Ser107Thr) mutation and were classified as Clade 2b.4. Recombination analysis identified 32 putative recombination events, including both inter- and intra-clade types. Genes with diverse functions are under differential selective pressures, with 3–20 positively selected sites detected in *ORF17*, *ORF33*, *ORF33.5*, and *ORF14* (gC). These findings on new subclades, frequent recombination, and rapidly changing genes crucial for viral adaptation are important for controlling future outbreaks and improving vaccine effectiveness. The research provided critical resources for investigating VZV genomic evolution in Beijing and to offer new insights into viral evolution and transmission patterns for public health initiatives.

## Introduction

The varicella-zoster virus (VZV), classified as Human Herpesvirus 3, is a neurotropic α-herpesvirus transmitted *via* respiratory droplets and direct contact. It causes two clinically distinct diseases: varicella (chickenpox) and herpes zoster (shingles) (Zerboni et al. 2014). Varicella predominantly affects school-aged children, often leading to seasonal epidemics; while typically self-limiting, severe complications may arise in immunocompromised individuals. Herpes zoster arises from the reactivation of latent VZV in sensory ganglia, commonly associated with age-related immune decline, and presents as dermatomal vesicular eruptions. Postherpetic neuralgia, a chronic pain syndrome following acute herpes zoster infection, currently lacks a universally effective treatment (Tang et al. 2023). Although live-attenuated vaccines derived from the vOka strain (Ozaki and Asano 2016) have significantly reduced disease burden globally (Shaw and Gershon 2018), breakthrough infections persist. Critically, both wild-type and vaccine strains could establish latency in neural ganglia (Quinlivan and Breuer 2014). Co-infection with multiple strains enables homologous recombination through genetic exchange, a key evolutionary driver in herpesviruses (Jensen et al. 2020). Recent studies demonstrate frequent inter- and intra-clade recombination events in VZV (Zell et al. 2012, Pontremoli et al. 2020), raising concerns about altered viral pathogenicity in recombinant strains and potential impacts on vaccine efficacy—factors that may influence public health strategies and vaccination adherence.

The VZV genome contains linear double-stranded DNA (~125 kb, 46% GC content) organized into unique long (UL) and short (US) segments flanked by terminal (TRL/TRS) and internal (IRL/IRS) repeats, encoding 71 open reading frames (ORFs) (Cohen 2010). Five genomic repeat regions include three within coding regions (R1–R3) and two non-coding repeats (R4–R5) (Depledge and Breuer 2023). Prior to the advent of whole-genome genotyping, various single nucleotide polymorphism (SNP)-based genotyping methods for VZV classification caused confusion in strain discrimination; however, these methods were still widely applied (Barrett-Muir et al. 2001, Faga et al. 2001, Loparev et al. 2004). Previous studies determined seven major clades based on the whole-genome sequences (Breuer et al. 2010): Clades 1 and 3 predominantly circulate in North America and Europe, while Clade 4 exhibits a global distribution. Clade 5 remains the only African-endemic genotype (Schmidt-Chanasit and Sauerbrei 2011), whereas Clades 6 and 9, identified in Southern California, represent recently discovered recombinant clades (Norberg et al. 2015, Jensen et al. 2017). Notably, both the parental (pOka) and vaccine (vOka) strains belong to Clade 2, in which viral sequences from Asian and North American populations occupy phylogenetically divergent nodes, suggesting region-specific evolutionary trajectories (Depledge and Breuer 2023). Furthermore, China’s complex VZV epidemiology includes recent reports of recombinant strains from Clades 1 and 2 (J. Li et al. 2024a). While conventional recombination-detection methodologies have primarily relied on sequence alignment to infer recombination events, the high genetic conservation across VZV clades, combined with large-scale genomic datasets, poses significant challenges for accurate recombination detection and precise breakpoint localization. Emerging evidence suggests that positively selected non-core herpesvirus genes mediate immune evasion through evolutionary adaptations. For instance, host-driven selection pressures have driven functional diversification of ICP47 in herpes simplex virus types 1 and 2 (HSV-1/HSV-2), attenuating its immunosuppressive activity (Mozzi et al. 2022). Although evolutionary mechanisms are well characterized in alpha-herpesviruses, comprehensive analyses of analogous VZV immunomodulatory networks remain limited, highlighting critical gaps in understanding VZV-host coevolution. Moreover, the scarcity of whole-genome sequencing data limits systematic investigations of viral evolutionary dynamics, emphasizing the urgent need for expanded genomic studies to elucidate current molecular epidemiological trends in VZV.

This study presents whole-genome sequencing and phylogenetic analysis of 25 VZV strains from Beijing, China. All strains clustered within Clade 2, with subclade analysis identifying 2b.4 as the predominant subclade based on characteristic variations. For the first time, the recombination detection tool CovRecomb was applied to VZV, revealing 32 putative recombination events, including both inter- and intra-clade exchanges. Among genes exhibiting dN/dS > 1, we identified 3 to 20 positively selected sites in *ORF17*, *ORF33*, *ORF33.5*, and *ORF14*. These findings systematically elucidate the molecular epidemiology and evolutionary dynamics of circulating VZV strains in Beijing, providing a theoretical foundation for optimizing vaccine design and therapeutic interventions.

## Materials and methods

### Participants and sample collection

Samples were collected from 24 patients with chickenpox and four with herpes zoster collected in Beijing Di’tan and You’an Hospital between March 2023 and March 2024. All samples were confirmed using fluorescence quantitative PCR (qPCR) (*N* = 28). The collected vesicular fluid samples were then subjected to sequencing. The Ethics Committee of Beijing Di’tan Hospital approved the study protocol (Approval No. DTEC-KY2021–058-01), and written informed consent was obtained from all patients or their family members.

### Library construction and sequencing

The mNGS library construction and genome sequencing were performed as previous studies (Y. Chen et al. 2023; Wu et al. 2024). In brief, DNA extraction was performed using the QIAamp DNA Mini Kit (Qiagen, USA) in accordance with the manufacturer’s protocol. Libraries were constructed using the Nextera XT DNA Sample Preparation Kit (Illumina) and purified with AMPure XP Beads (Becmkan). Library fragments were verified using an Agilent 2100 Bioanalyzer (Agilent). Following quality control, sequencing was performed on an Illumina NovaSeq 6 000 platform.

### Dataset construction

Raw sequencing data were processed using Fastp (v0.23.2) (version 0.23.2) (S. Chen et al. 2018) to filter low-quality reads. BWA (version 0.7.17) (H. Li and Durbin 2009) was employed to map clean reads to the human reference genome (hg38), and host-derived reads were excluded. Genomic positions with ≥10× sequencing coverage in ≥98.0% of nucleotides were retained. Three samples with low coverage (Sample IDs: BJ2023017, BJ202320, and BJ202404) were removed from downstream analysis. Integrative Genomics Viewer (Robinson et al. 2011) was used to viewed reads alignments. The paired-end reads were aligned to the reference genome using the minimap2 (H. Li 2018) tool and subsequently processed and sorted with samtools (Danecek et al. 2021) view and samtools sort. Variant detection was carried out using FreeBayes (Garrison and Marth 2012), the bcftools command was applied to quality-filter variants. and mutations were annotated using SnpEff v5.2a (Cingolani et al. 2012). The data reported in this paper have been deposited in the GenBase (46) at National Genomics Data Center (47), Beijing Institute of Genomics, and Chinese Academy of Sciences /China National Center for Bioinformation, under accession number C_AA107080.1 to C_AA107104.1 (Table S4).

### Phylogenetic and selection analysis

A total of 158 publicly available VZV genomes (with > 99% nucleotide coverage relative to the reference strain) were downloaded from the NCBI GenBank database (as of 1 October 2024). After excluding artificial recombinant viruses and wild-type strains subjected to extensive passaging, the final dataset comprised 183 VZV genomes (Table S5). Sequences were aligned using MAFFT (version 7.487) (Katoh and Standley 2013), and a maximum likelihood (ML) tree was constructed using IQ-TREE (version 2.3.6) (Nguyen et al. 2015), with the general time-reversible (GTR) nucleotide substitution model. Node support was evaluated using 1 000 bootstrap replicates. ML trees were visualized using Interactive Tree of Life (iTOL; https://itol.embl.de/). We employed the NeighborNet algorithm within SplitsTree4 software (Huson and Bryant 2006) to construct a phylogenetic network after removing terminal repeat regions and gaps. For SNPs analysis, all datasets were compared with the Clade 1 reference genome (Dumas strain VZV; accession: NC_001348.1), utilizing NextClade (Hadfield et al. 2018) to identify nucleotide and protein mutations, Clade-specific mutation sites were visualized with Snipit (https://github.com/aineniamh/snipit). The dN/dS (ω) ratios were calculated using the yn00 program in PAML (Yang 2007). Coding sequences (CDS) from the VZV reference genome were concatenated, and the same program was used to estimate the total number of nonsynonymous (N) and synonymous (S) sites genome wide. Positive selection was assessed using EasyCodeML (Gao et al. 2019) under the site model. Phylogenetic trees were reconstructed under the GTR model with 1 000 bootstrap replicates and universal codon tables. Initial M0 versus M3 comparisons indicated significant site heterogeneity, while M1a versus M2a and M7 versus M8 analyses confirmed positively selected sites (ω ≥ 1). Protein domains were predicted using InterPro (Blum et al. 2025), and structures were modelled with AlphaFold (Harris et al. 2020). PyMOL (Schrödinger and DeLano (2020) was employed to analyse hydrogen bond and hydrophobicity changes post-mutation. The functional impact of amino acid substitutions was evaluated using PROVEAN (Choi and Chan 2015).

### Recombination identification

Sequences from Clade 2 were trimmed to remove terminal repeat regions and gaps, followed by the construction of a haplotype network using fastHaN (63) and visualization with HapNetworkView software (64). We applied the recombination detection tool CovRecomb, developed by Li et al., to analyse recombination events in VZV (22). Briefly, we aligned all 183 complete VZV genomes with the reference Dumas strain using Nextclade, converting the genomes into a set of single-nucleotide polymorphisms (SNPs). For each clade, we extracted mutations shared by > 80% of clade members to construct a clade-definition library containing representative clades and their characteristic mutations. Additionally, we identified characteristic mutations within the five subclades of Clade 2 while analysing intra-clade recombination events. The mutation vector of target samples was mapped to a predefined lineage-paired score matrix to calculate the hypergeometric distribution score (raw *P*-value) for each clade pair. We then applied the Bonferroni correction to adjust the scores (corrected *P*-value) and minimize false positives. Clade pairs with the lowest corrected *P*-value (*P*<.05) were selected as the best candidates and further assessed as putative recombinants based on four biological criteria: (i) a restricted number of breakpoints (≤4) in the combined characteristic mutations, accounting for the VZV genome’s high conservation and larger size compared to SARS-CoV-2; (ii) at least five consecutive characteristic mutations in both parental clades; (iii) characteristic mutations from the two parental clades not co-occurring in the most probable non-recombinant clade; and (iv) no single genome implicated in the recombination event. For intra-clade mutation detection, due to higher sequence similarity, we relaxed the requirement to at least two consecutive characteristic mutations in both parental clades.

### Statistical analysis

All datasets were analysed using R v4.0.2 (https://www.r-project.org) and visualized using the ggplot2 package (https://cran.r-project.org/package=ggplot2). To examine the distribution of all mutations within the VZV genome, principal coordinate analysis (PCoA) was performed using a Bray-Curtis distance matrix. Differences in viral composition among clades were assessed for statistical significance using the adonis function from the vegan package (https://cran.r-project.org/package=vegan), which performs non-parametric multivariate analysis of variance (ANOVA) based on Bray–Curtis distances with 999 permutations. For group comparisons, one-way ANOVA was applied, while within-group differences were evaluated using Student’s *t*-test. All visualizations were generated using the ggpubr package (https://rpkgs.datanovia.com/ggpubr/). Significance thresholds were defined as follows: ^*^P≤.05, ^**^P≤.01, ^***^P≤.001, ^****^P≤.0001 ns = not significant.

## Results

### Characterization of circulating VZV strains in Beijing

Clinical specimens were collected from 24 varicella and 4 herpes zoster patients at Beijing Di’tan and You’an Hospital between March 2023 and March 2024. Clinical demographic analysis showed that varicella patients ranged in age from 3 months to 14 years (male-to-female ratio: 4:3), with no discernible epidemiological connections among cases. Herpes zoster patients had a mean age of 39.7 years (male-to-female ratio: 1:1), and none had received varicella-zoster vaccination (Table S1). VZV-positive samples were confirmed *via* qPCR. Strains with incomplete metadata or genome coverage below 98% (at 10× sequencing depth) were excluded, yielding 25 complete genome sequences.

### VZV strains identified in Beijing predominantly belong to clade 2b.4

**Figure 1:**
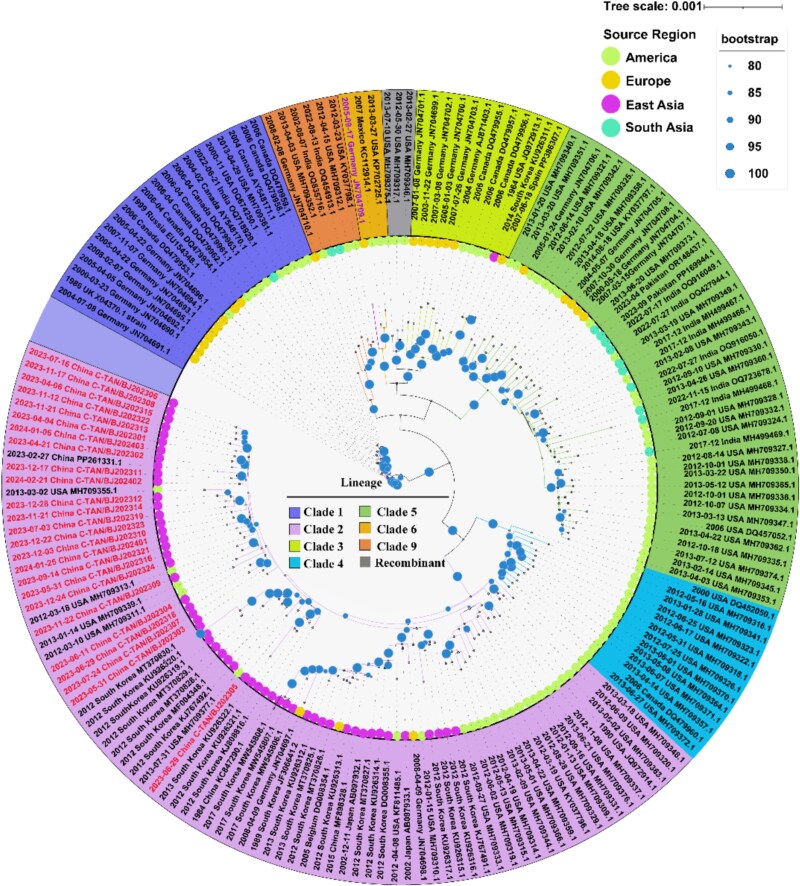
Maximum likelihood phylogenetic tree of VZV whole genome sequences. Phylogenetic clades were differentiated using chromatic zonation along the concentric periphery of the evolutionary tree. The geographical origins of the sequences were indicated in the inner ring through a coding system, with Beijing strains specifically marked and global strains distinguished separately. Clade VIII (a provisional clade, identified by distinctive tip labels), clusters phylogenetically with Clade 6, while the designated sector denotes uncharacterized recombinant sequences.

To characterize VZV genomic diversity in Beijing, we integrated newly sequenced genomes with 158 publicly available ones. The maximum-likelihood phylogenetic tree constructed from these genomes indicated that all Beijing strains clustered within Clade 2 (Fig. 1), consistent with previously reported prevalent strains in China (Xu et al. 2016). Phylogenetic network analysis further validated these findings (Fig. S1).

**Figure 2:**
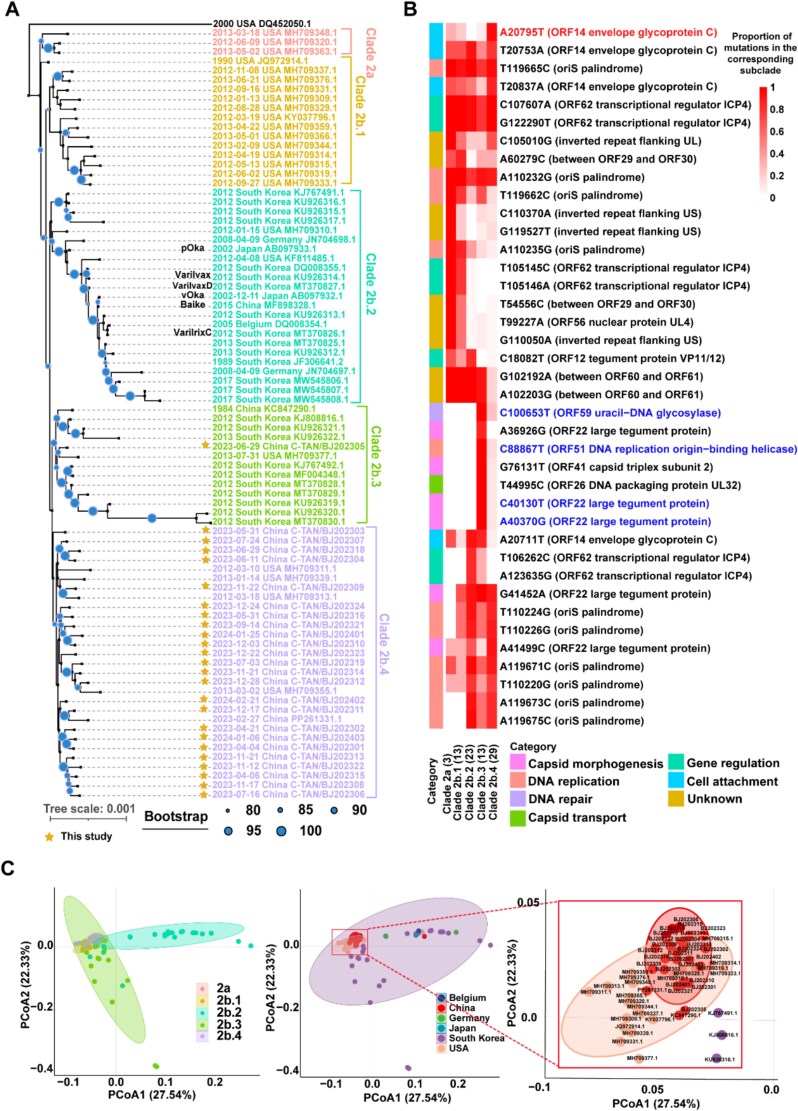
Molecular genetic evolutionary characteristics of clade 2. (A) Clade 2 was subdivided into five subclades. Strains generated by this study are highlighted with yellow pentagons, while vaccine strains are annotated with their common names in text. DQ452050.1 (clade 4) served as the outgroup in the phylogenetic analysis. (B) A heatmap illustrating the proportional distribution of feature mutations across clade 2 subclades. Mutation site 20 795 (specifically highlighted) is predominantly clustered in clade 2b.4. Feature mutations (distinctively highlighted) of clade 2b.3 from recombinant sequences. (C) PCoA revealed phylogenetic divergence between clade 2 subclades and their geographic distributions. An enlarged panel provides a detailed visualization of genetic distance patterns among sequences from Beijing.

Based on characteristic variants, Clade 2 was subdivided into five subclades: 2a, 2b.1, 2b.2, 2b.3, and 2b.4 (Fig. 2A). The Clade 2-specific phylogenetic network confirmed this classification (Fig. S2), with subclade-defining variants identified after excluding those shared across Clade 2 (Fig. 2B). Variants at Clade 2a (C110370A, G119527T), Clade 2b.2 (T106262C, A123635G), Clade 2b.3 (A36926G, A40130G, A40370G, T44995C, G76131T, C88867T, C100653T), and Clade 2b.4 (A20795T), distinguish these branches (Table S2). The principal coordinate analysis (PCoA) of Clade 2 sequences revealed greater dispersion in subclades 2b.2 and 2b.3 compared to the tightly clustered 2b.4 strains (Fig. 2C). Geographically, subclades 2a and 2b.1 were predominantly found in the United States, 2b.2 and 2b.3 were mainly distributed in Japan and South Korea, and 2b.4 circulated in both China and the United States.

### Both inter- and intra-clade recombination have contributed to VZV evolution

**Figure 3:**
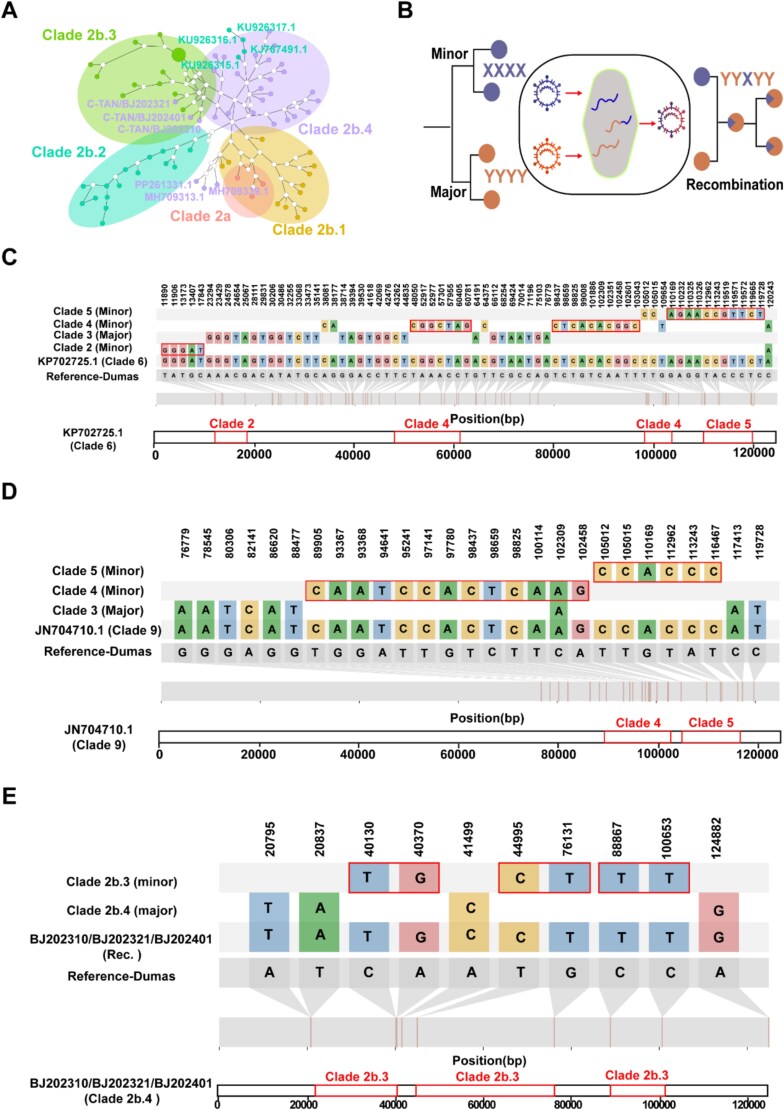
Whole-genome sequences of VZV reveal extensive inter- and intra-clade recombination. (A) A haplotype network diagram of clade 2 subclades, with distinct colours representing individual branches. Outlier strains suggest potential recombination events, and non-clustering haplotypes are explicitly labelled. The dots represent internal nodes within the haplotype network. (B) The recombinant viral strain YYXYY incorporates diagnostic mutations from both parental clades, with nucleotide motifs YYYY and X tracing to its major and minor parental clades, respectively. (C) The KP702725.1 (clade 6) strain identifies clade 3 as the major parent, with clades 2, 4, and 5 as minor contributors. (D) The JN704710.1 (clade 9) strain similarly designates clade 3 as the major parent and clades 4 and 5 as minor contributors. (E) Three Beijing strains (BJ202301, BJ202321, and BJ202401) were identified as putative recombinants, with clade 2b.4 and 2b.3 serving as the major and minor parents, respectively. The rectangles highlight diagnostic mutations in recombinant regions along with their genomic positions.

Genomic recombination, a well-documented mechanism driving herpesvirus evolution (Norberg 2010), may have contributed to the emergence of specific VZV clades (Norberg et al. 2015). Haplotype network analysis revealed interconnectivity among the subclades of Clade 2, suggesting potential recombination events (Fig. 3A). Notably, during recombination events between two clades, clade-feature variants within genomic segments are co-inherited (Fig. 3B).

We adapted recently published methods for detecting SARS-CoV-2 recombination (J. Y. Li et al. 2024b) to analyse recombination events in VZV. Applying this approach to recombination analysis of 183 whole-genome sequences identified 32 putative recombination events (Table S3). For instance, strain KP702725.1 (Clade 6) originated from a complex recombination process involving parental Clade 2, 3, 4, and 5 (Fig. 3C). Similarly, strain JN704710.1 (Clade 9) exhibited a hybrid genomic structure, with Clade 3 as the major parental lineage and Clades 4 and 5 as minor contributors (Fig. 3D). Additionally, we detected Clade 3-derived genomic fragments within sequences phylogenetically assigned to Clade 5 (Fig. S3A). After validating the methodology, we further examined three unclassified sequences (grey zone) within the phylogenetic tree, along with one sequence provisionally designated as Clade VIII (purple label) (Fig. 1). Strains MH709317.1, MH709375.1, and MH709346.1 (temporarily designated Clade 3X) along with Clade 3-associated strain JN704701.1 were identified as potential recombinants, with Clade 3 as the major parental lineage and Clade 2 and 5 as minor contributors (Fig. S3B). Clade 3X potentially represents novel evolutionary subclades or a recombinant clade. Further analysis of Clade VIII confirmed Clade 3 as the major parental lineage, with Clades 2 and 4 as minor contributors (Fig. S3C).

The sequenced strains of Beijing were subjected to intra-clade analysis. Due to high intra-clade sequence similarity, detection parameters were adjusted to require ≥2 consecutive variants for recombination identification. While this increased sensitivity, it also raised the risk of false positives. By integrating subclade-defining variants and haplotype network analysis, we excluded most false positives and identified three sequences (C-TAN/BJ202401, C-TAN/BJ202310, C-TAN/BJ202321) as putative intra-clade recombinants (Fig. 3E). These strains harboured Clade 2b.3-derived fragments, including regions spanning nucleotides 44 995–76 131 and 88 867–100 653, as well as a fragment adjacent to the R3 region (20637–41 499). Recombinants in other subclades annotated within the haplotype network were also characterized (Table S3).

### VZV genes exhibit substantial heterogeneity in evolutionary selection

**Figure 4:**
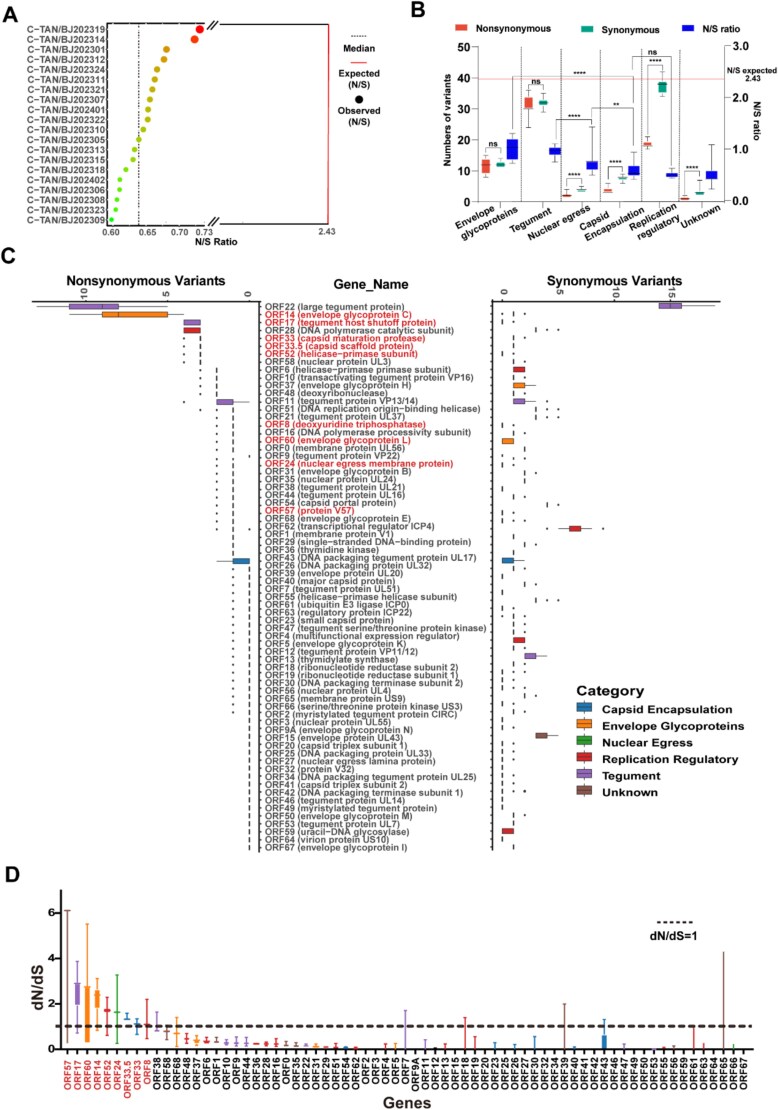
Evolutionary variant characteristics of SNPs in the genome. (A) the observed nonsynonymous-to-synonymous (N/S) ratio for 25 whole-genome sequences from Beijing, with bubble size proportional to the ratio magnitude. The median and expected N/S values are indicated by a dashed line and a solid line, respectively. (B) Synonymous and nonsynonymous variants alongside N/S ratios for the same 25 sequences, categorized by functional class. The left y-axis represents variants, while the right y-axis shows ratios, with the expected N/S value marked by a solid line. (C) Synonymous and nonsynonymous variants per open reading frame (ORF) across the 25 sequences. Gene products are labelled in parentheses and colour-coded by functional category, with genes exhibiting dN/dS > 1 highlighted. (D) The dN/dS ratio for each ORF was calculated by comparing varicella-zoster virus (VZV) clinical strains to the reference genome. A pseudocount threshold (0.001) was applied to dS to avoid division errors. The dashed line denotes the neutral evolution threshold (dN/dS = 1), and genes with dN/dS > 1 are highlighted.

We performed statistical analysis on variants in the 25 sequences collected in Beijing (Fig. S4). Relative to the Dumas reference genome, the C-TAN/BJ202401 strain exhibited the highest number of variants (*n* = 236, including 215 SNPs), whereas C-TAN/BJ202316 harboured the fewest (*n* = 184, 167 SNPs). The observed nonsynonymous (N) to synonymous (S) substitution ratios among the strains ranged from 0.57 to 0.73 (median = 0.63). The expected N/S ratio for VZV genes (N/S_expected_ = 2.43) was substantially larger than the observed values (Fig. 4A).

**Figure 5:**
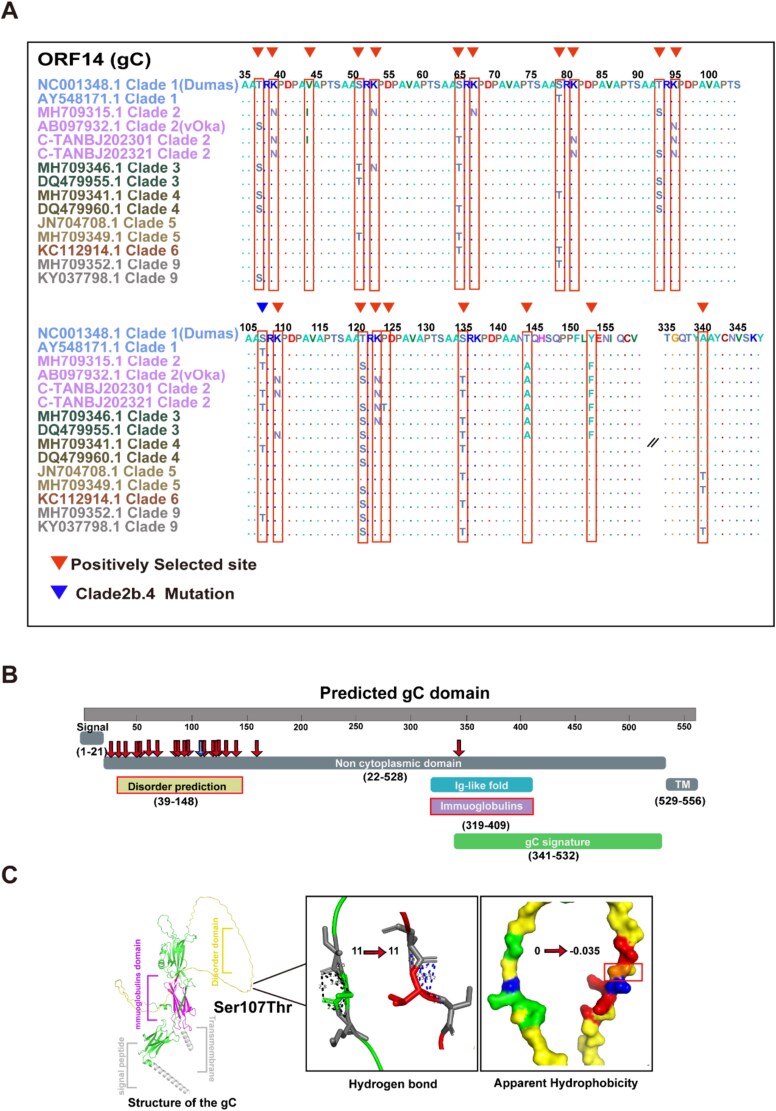
Mutations at selective sites facilitate the adaptive evolution of gC. (A) Analysis of positively selected sites in representative strains from each clade. Positively selected sites in the *ORF14* gene are marked with triangles and rectangles, while the distinct triangle indicates the enriched mutation site 20 795 identified in clade 2b.4. (B) Structural domains of the gC protein were predicted using an online domain-prediction tool. The downward arrows denote positively selected sites, and the distinct arrows represent mutation sites enriched in clade 2b.4. (C) The three-dimensional structure of the gC protein, predicted using AlphaFold, displays the S107T mutation (gene position 20 795) in clade 2b.4, disordered regions, immunoglobulin domains, and signal peptide and transmembrane (TM) regions. Changes in hydrogen bond interactions and hydrophobicity resulting from mutations between gC and gC: S107T were analysed and visualized using PyMOL. Numerical values indicate alterations in hydrogen bond counts and hydrophobicity, with the rectangle highlighting regions of hydrophobic environment changes.

Selection pressure varies among genes from different functional categories in SARS-CoV-2 (González-Vázquez and Arenas 2023) and other DNA viruses (Suzuki and Gojobori 2001; Bao et al. 2021). We classified viral genes into those involved in capsid encapsulation, envelope glycoproteins, nuclear egress, replication regulation, tegument assembly, and unknown functions. Synonymous mutations significantly outnumbered nonsynonymous mutations in replication regulation genes (Student’s t-test, *P*<.0001). The nonsynonymous-to-synonymous (N/S) ratio was also larger in tegument and envelope glycoprotein genes relative to replication and capsid encapsulation genes (one-way ANOVA, *P*<.0001; Fig. 4B, Table S10). Analysis of synonymous and nonsynonymous variants across viral genes revealed *ORF22* as the most genetically diverse, with *ORF14* also displaying a high frequency of nonsynonymous variations. The CDS of 18 genes are highly conserved and lack nonsynonymous variation sites. Consequently, these genes were excluded from subsequent selection site analysis (Fig. 4C). Evolutionary analysis using dN/dS ratios identified selective pressures in genes from the Beijing viral strains. Nine ORFs (8, 14, 17, 24, 33, 33.5, 52, 57 and 60) showed evidence of positive selection (dN/dS > 1; Fig. 4D). Positively selected sites were identified using strains with complete CDS (*n* = 139). While *ORF8*, *ORF24 ORF52*, *ORF57*, and *ORF60* lacked statistically supported adaptive sites, sites under positive selection were detected in *ORF14*, *ORF17* and *ORF33*/*ORF33.5* (Fig. 5A, Fig. S6A-B, and Table S6–S9).

### Hotspot mutations in Beijing VZV strains lack function-associated signatures

The *ORF14* (gC, glycoprotein C) contained twenty positively selected sites (T37S, K39N, V44I, S51T, K53N, S65T, K67N, S79T, K81N, T93S, K95N, S107T, K109N, T121S, K123N, P124T, S135T, T144A, Y153F and A340T; Fig. 5A, Table S7). The mutation in *ORF14* (S107T), which enriched in subclade 2b.4, was also identified as a positively selected site. InterPro domain analysis mapped eighteen adaptive gC sites (T37S, K39N, V44I, S51T, K53N, S65T, K67N, S79T, K81N, T93S, K95N, S107T, K109N, T121S, K123N, P124T, S135T and T144A) to intrinsically disordered regions, while the A340T site localized to the immunoglobulin-binding domain (Fig. 5B). AlphaFold2 structural predictions indicated preserved hydrogen bonding (*n* = 11) at position 107 (Ser > Thr), but molecular surface analysis revealed increased hydrophobicity (WT: 0 versus Mut: −0.035; Fig. 5C), suggesting potential alterations in interaction interfaces.

Alignment analysis of the major antigenic and drug-target protein sequences in the Beijing strain, compared to the vOka strain, identified mutations in gE (I525S), gB (I593V), and gH (A500V, A477V). None of these mutations occurred within known antigenic epitopes (Table 1). Additionally, T219P and R251K mutations were detected in the acyclovir-targeted DNA polymerase (Table 2). PROVEAN protein function analysis predicted neutral effects for these variants, with no documented clinical drug resistance.

**Table 1:** Amino acid substitutions in the glycoproteins gE, gB, and gH between wild-type strains and the vOka vaccine strain

**seqName**	**gB**	**gH**	**gE**	**Provean** **predict**
C-TAN/BJ202302	I593V	–	–	neutral
C-TAN/BJ202401	–	A500V	I525S	neutral
C-TAN/BJ202315	–	A477V	–	neutral

Amino acid mutations divergent from the vOka strain.

**Table 2:** Amino acid substitutions in DNA polymerase between wild-type strains and the vOka vaccine strain

**seqName**	**DNA polymerase**	**Provean** **predict**
C-TAN/BJ202302	R251K	Neutral
C-TAN/BJ202307	T219P	Neutral
C-TAN/BJ202304	T219P	Neutral
C-TAN/BJ202301	R251K	Neutral
C-TAN/BJ202318	T219P	Neutral
C-TAN/BJ202324	T1091I	Neutral
C-TAN/BJ202403	R251K	Neutral

Amino acid mutations divergent from the vOka strain.

## Discussion

A total of 25 VZV strains from Beijing and 158 publicly sequences were analysed. Phylogenetic tree and network revealed that all Beijing strains belong to Clade 2, consistent with prior reports of Clade 2 dominance in China (Wang et al. 2024). Further subdivision of Clade 2 into five subclades showed that 24 Beijing strains clustered with one southern Chinese strain and four United States strains into subclade 2b.4. Clinical metadata for the United States strains indicated that 3 of 4 patients were born in Asia (Jensen et al. 2020). Recombination analysis identified widespread inter- and intra-clade recombination events, highlighting their role in increasing genetic diversity of VZV. Genes with distinct functions were subject to differential selection pressures. Additionally, we identified an enriched mutation at position 20 795 (S107T) in *ORF14* within subclade 2b.4. As a positively selected site, structural prediction suggests this mutation alters residue hydrophobicity, potentially affecting protein interactions. Genomic data for VZV, particularly within Clade 2, remain underrepresented in China (only three strains). Our study substantially expanded this dataset (by a factor of 8.33), and revealed unique evolutionary characteristics of the virus.

The investigation of VZV recombination reveals multiple evolutionary mechanisms driving viral diversification. We identified inter-clade recombination events contributing to the formation of new clades, including the hybrid genomic architectures of Clades 6 and 9, and detected potential ancestral recombination between donor sequences from Clades 3 and 4 within the ancestral lineage of Clade 5 (Jensen et al. 2020). Three sequences (MH709317.1, MH709375.1, and MH709346.1) were identified as recombinants derived from the same putative recombination event. Recombinant analysis revealed their major parental inherited from Clade 3, suggesting their classification as a novel clade (Clade 3X). Notably, the JN704701.1 sequence previously classified under Clade 3 was also identified as a recombinant for the first time. Recombination analysis suggested that Clade 3 serves as the major parental lineage for multiple clades. Subsequently, phylogenetic analysis of core conserved genes from Simian Varicella Virus and VZV indicated that Clade 3 may represent the ancestral clade (Fig. S5). Additionally, we documented the first evidence of intra-clade recombination within subclade 2b.3 through systematic screening of strains C-TAN/BJ202310, C-TAN/BJ202321, and C-TAN/BJ202401. Importantly, phylogenetic tracing excluded vaccine strain (Clade 2b.2) involvement, consistent with the absence of varicella vaccination history in all subjects. These results corroborate the findings of haploid evolutionary relationships and the feature mutations in subclades. However, our reference-dependent approach using the Dumas strain (Clade 1) introduced detection bias, preventing identification of Clade 1 recombinant fragments—a systemic limitation underscoring challenges in characterizing recombination events involving the reference clade. Collectively, these findings establish dual recombination mechanisms (inter- and intra-clade) as important drivers of VZV evolution. The recurrent of ancestral and contemporary recombination events emphasizes the critical need for heightened vigilance toward recombination-induced discrepancies when conducting variants surveillance and genotyping of VZV.

By analysing the evolutionary dynamics of VZV, we identified distinct selection patterns across viral genes. Whole-genome sequencing of Beijing-derived VZV strains revealed a significantly lower nonsynonymous-to-synonymous (N/S) substitution ratio in replication-essential genes, such as those encoding DNA polymerase and helicase, indicating strong purifying selection. This suggests evolutionary constraints to maintain core replication fidelity. In contrast, replication regulatory genes exhibited the highest frequency of synonymous site changes, which may facilitate viral evolution by optimizing codon usage or RNA secondary structures (Jordan-Paiz et al. 2021, Liu et al. 2021, Khandia et al. 2023, Rad et al. 2023). Meanwhile, tegument proteins displayed an enrichment of nonsynonymous substitution sites, suggesting adaptive plasticity (McGeoch et al. 2006, Rathbun and Szpara 2021), particularly in host interaction domains such as STAT3/NF-κB modulation regions (Jones and Arvin 2006). Gene-specific analyses identified *ORF22* as the most polymorphic locus, with both synonymous and nonsynonymous variations. Notably, nonsynonymous variants clustered near the pUL25-binding domain (an HSV-1 UL36 homologue), potentially affecting capsid transport and assembly mechanisms (Ivanova et al. 2016). Additionally, *ORF17* exhibited one of the highest dN/dS ratios, with positively selected sites in functional domains critical for mRNA cleavage and immune evasion (Sato et al. 2002, He et al. 2020), highlighting these regions as potential therapeutic targets.

Glycoprotein C mediates multifunctional signal integration by binding to diverse ligands. Structural prediction demonstrated a potential functional change in gC (*ORF14*), where the S107T mutation (Clade 2b.4) alters hydrophobicity within an intrinsically disordered region that may facilitate phase separation. This mutation in the protein also did not alter binding structure to relevant cytokines, such as CXCL13 and IFN-γ (Fig. S6C, Fig. S6D) (González-Motos et al. 2017, Jacobsen et al. 2024), due to challenges in establishing animal models and isolating VZV for cultivation, functional studies of VZV have primarily referenced the homologous model of HSV-1. Thus, the gC protein of HSV-1 may play a role in immune evasion mechanisms, despite only 34% sequence similarity between the two proteins (Grose 1991, Grose et al. 2010). The observed waning of vaccine efficacy through accumulating gE variants (*e.g.* North American D150N) demonstrating epitope erosion and enhanced virulence (Santos et al. 2000, Tipples et al. 2002). Although the major glycoproteins (gE, gB and gH) of VZV Beijing strains are highly conserved, alterations in minor glycoproteins and tegument proteins may compromise vaccine efficacy. Sequencing has detected no drug-resistant variants in Beijing; nevertheless, clinical sporadic cases warrant close monitoring (Hoffmann et al. 2017).

This study has several limitations that should be acknowledged. The analysis was constrained by a limited sample size and restricted temporal scope, which may diminish statistical power for detecting subtle evolutionary trends and limits the generalizability of population-level patterns. These limitations are further exacerbated by the lack of comparative phenotypic profiling across Clade 2 subbranches—while genomic divergence was characterized, critical biological parameters such as transmissibility, virulence, and immune evasion potential remain unassessed. Additionally, genetic signatures such as Clade 2b.4-specific gC protein substitutions and adaptive selection sites in structural proteins remain unvalidated. In the absence of functional assays, the biological consequences of these mutations on receptor tropism, antibody escape, or replicative fitness remain theoretical. In the geographical analysis of VZV phylogeny, low effective sample size (ESS) values (data not shown) were observed, potentially attributable to high recombination rates among strains. To address these gaps, four priorities emerge: (i) Expanding longitudinal sampling encompassing underrepresented regions to improve evolutionary pattern resolution; (ii) systematic phenotypic comparisons of Clade 2 subclades using *in vitro* and *ex vivo* models; (iii) mechanistic validation of candidate mutations *via* site-directed mutagenesis integrated with structural modelling to establish genotype–phenotype relationships; and (iv) functional prioritization of conserved genes. Such integrated approaches would address the current gap between observed genetic variation and its clinical-epidemiological relevance while enhancing predictive models of VZV evolution.

Finally, this study characterized the evolutionary dynamics of VZV in Beijing through whole-genome sequencing of 25 strains. The newly generated sequences and refined classification of Clade 2 subtypes enhance outbreak tracing, early warning systems, and viral evolution monitoring. Our findings demonstrate that the interplay between recombination and SNPs drives viral diversification, underscoring the necessity for sustained genomic surveillance to mitigate emerging public health threats. By enriching China’s VZV genomic database, this work provides critical insights to strengthen molecular epidemiology capacities and inform targeted prevention strategies.

## Supplementary Material

Supplementaryfigure_veaf076

Supplementarytable_veaf076

## Data Availability

Clinical samples and original records may be obtained from the corresponding author upon reasonable request, subject to approval from both Hebei Medical University and the China CDC. To request the data, you can contact the author *via* email at doctorhanxt@163.com and 90610101@hebmu.edu.cn
