# Supplementary material for: Decoding VZV’s evolutionary arsenal: how Beijing strains use recombination and adaptive mutations to thrive
Source: Virus Evol. 2025 Sep 23;11(1):veaf076. doi: 10.1093/ve/veaf076 (PMC12513170; doi:10.1093/ve/veaf076)
Supplement: Supplementaryfigure_veaf076 [file supplementaryfigure_veaf076.docx]

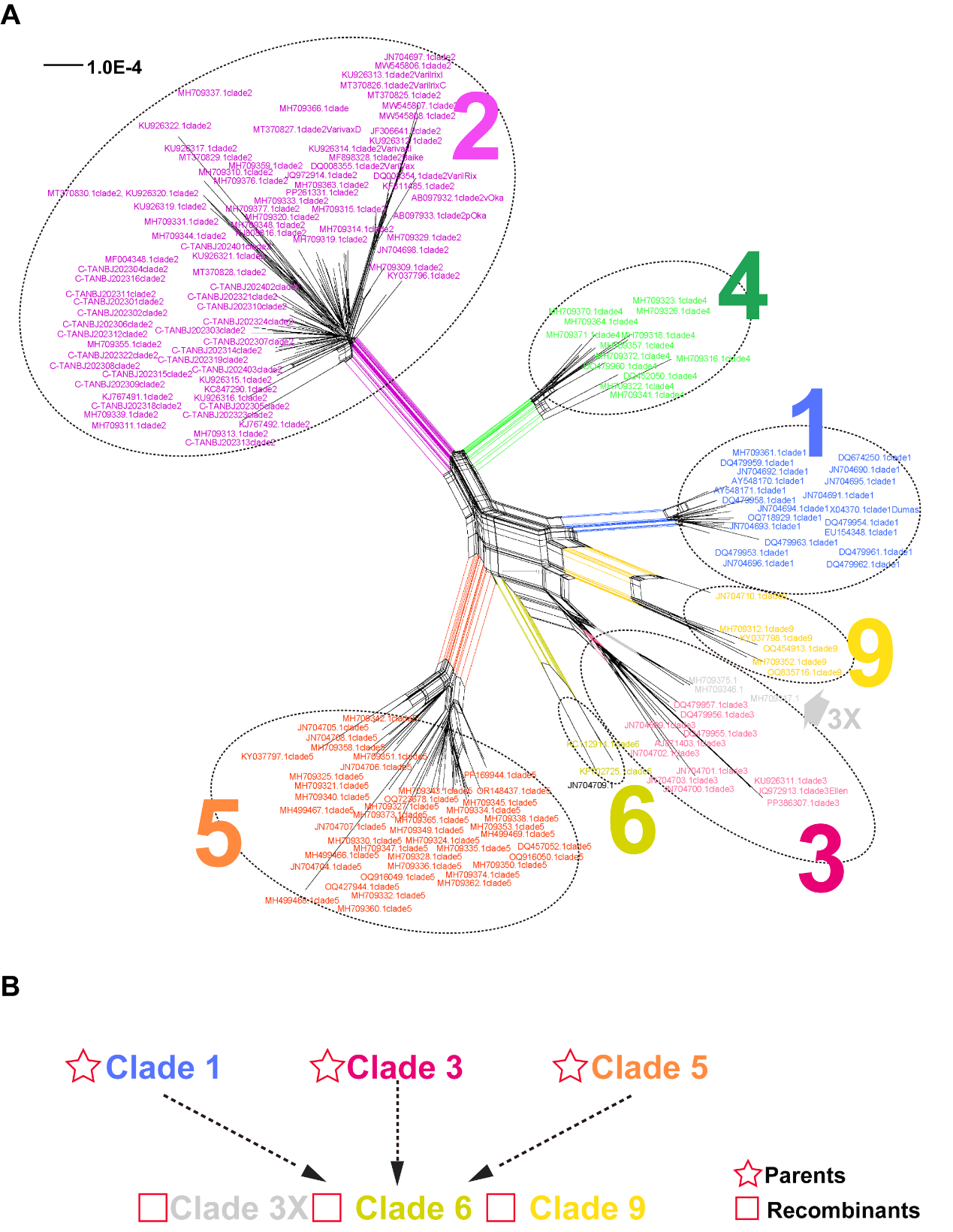


**Fig. S1 Phylogenetic neighbor-network analysis of 183 sequences.**

**(A)** Phylogenetic neighbor-network analysis of 183 sequences (Splitstree4). Colors represent distinct clades (consistent with the phylogenetic tree). The gray arrow points to the newly identified recombinant Clade 3X (this study; see Fig. S4B). **(B)** A visualization of the recombination relationships between clades, as revealed by the phylogenetic network.


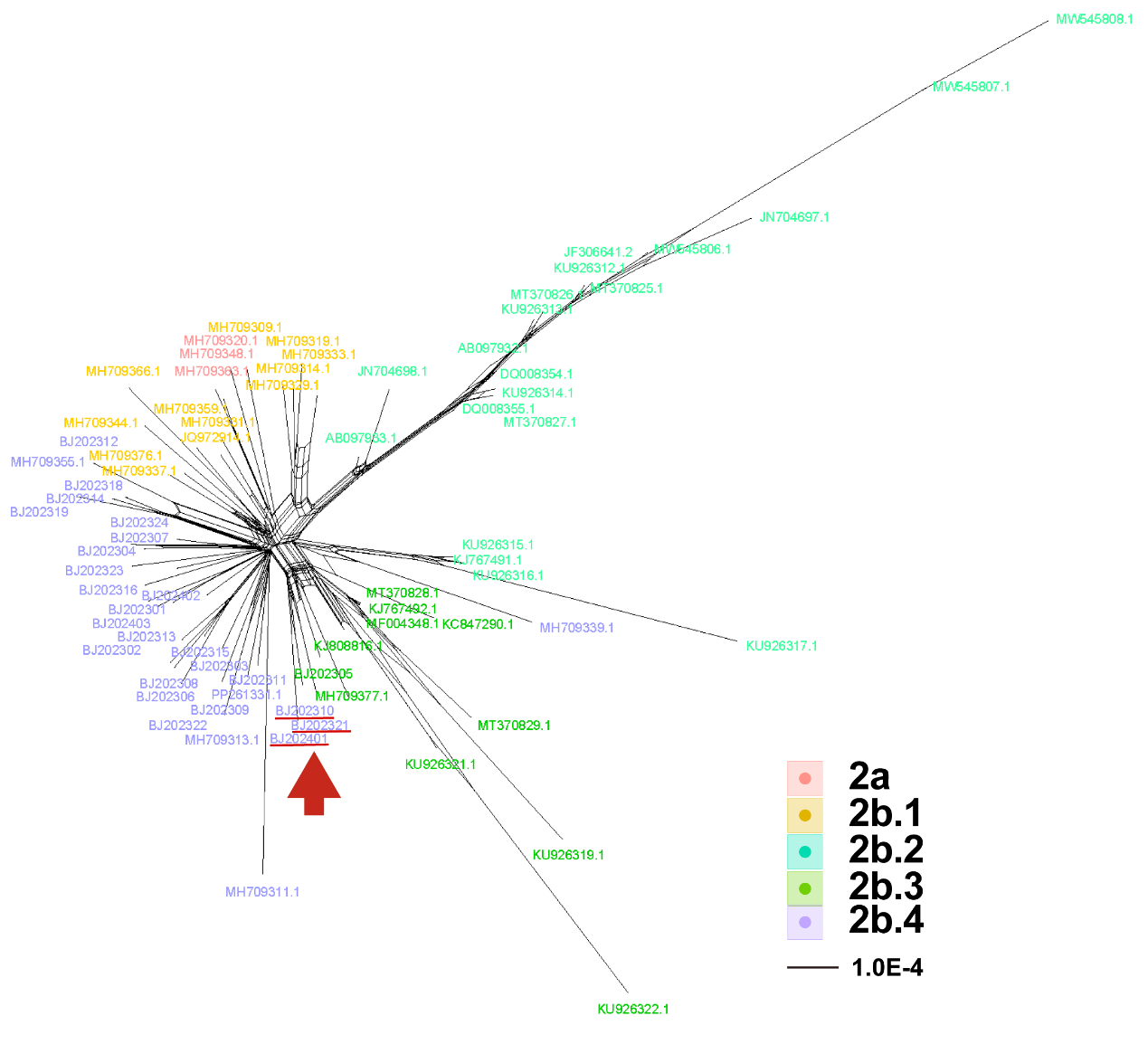


**Fig. S2 Phylogenetic neighbor-network of Clade 2 sequences.**

Phylogenetic neighbor-network of Clade 2 sequences (Neighbor-net, Splitstree4). Colors represent distinct subclades, consistent with tree and haplotype network analyses. The red arrow designates the three newly identified Beijing recombinant sequences (this study; as shown in Fig. 3E).

**
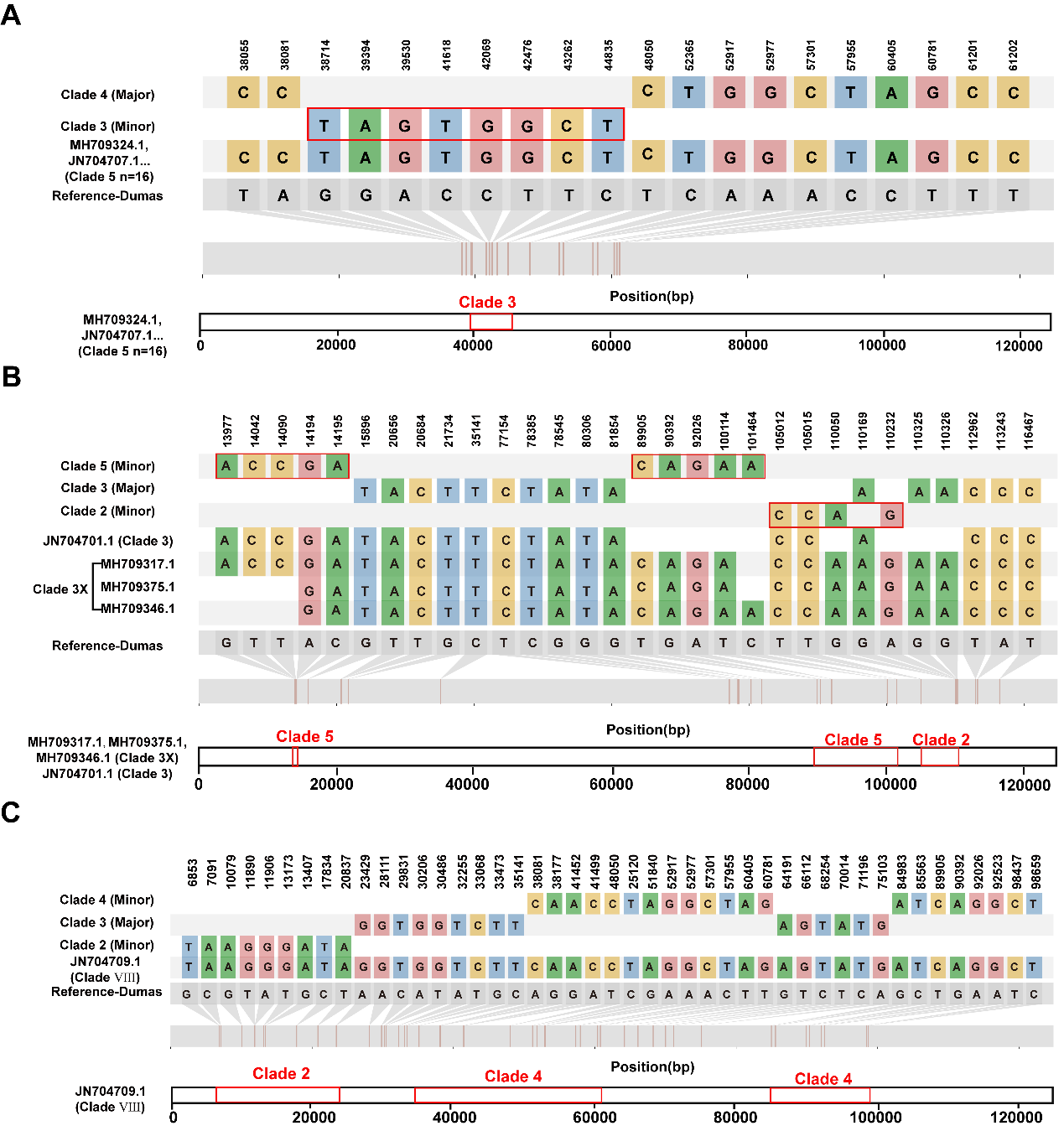
**

**Fig. S3 Identification of inter-clade recombinant sequences.** (**A**) Sixteen Clade 5 strains exhibit conserved recombination profiles, with Clade 4 serving as the major parental contributor and Clade 3 as the minor contributor; (**B**) Three strains (MH709317.1, MH709375.1, MH709346.1), tentatively designated Clade 3X, demonstrate recombination patterns involving the strain JN704701.1 (Clade 3). These events feature Clade 3 as the major parental lineage, supplemented by Clade 2 and 5; (**C**) The provisional strain JN704709.1 (Clade VIII) displays recombinant architecture with Clade 3 as the major parental source and Clades 2 and 4 as minor contributors. Red boxes annotate recombination-associated mutations and their genomic coordinates.


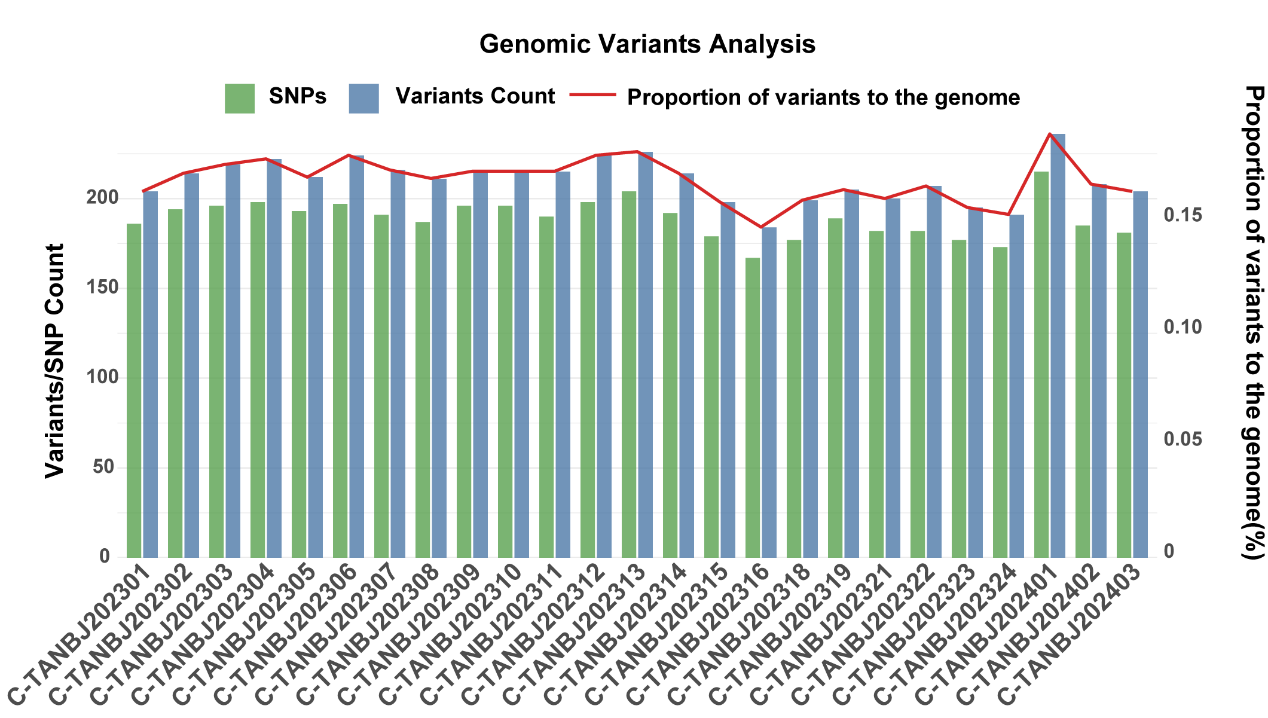


**Fig. S4 Variants analysis** **of the 25 sequences collected in Beijing.**

The count numbers of variants (blue bars) and SNPs (green bars) mapped to the genome from 25 sequences collected in Beijing, and the red line denotes the variant proportion (calculated as the number of variants divided by the total sequence length).


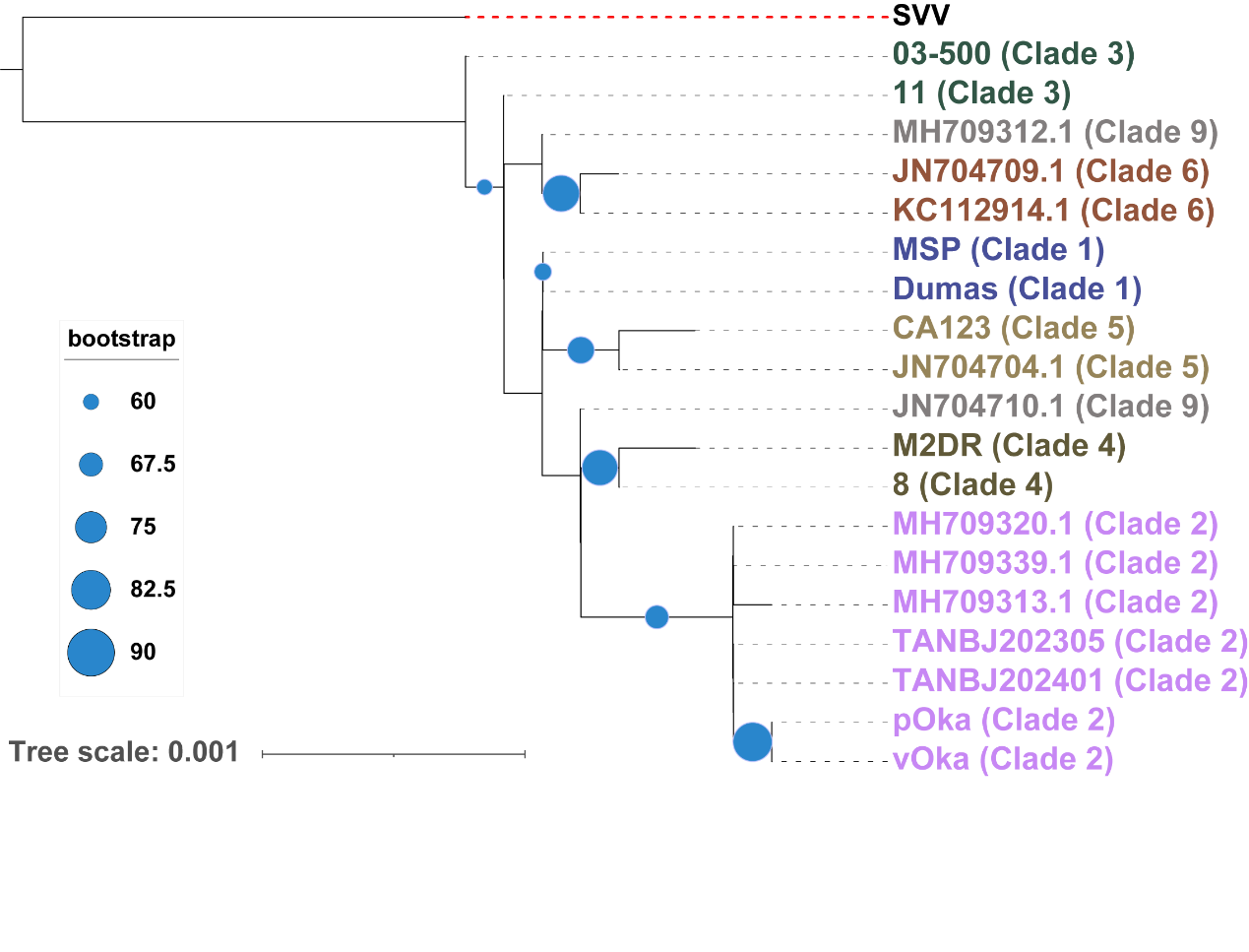


**Fig. S5 Incorporation of Simian Varicella Virus (SVV) in VZV Phylogenetic Analysis.**

A maximum-likelihood phylogenetic tree was constructed using conserved proteins ORF30-33, ORF40, and ORF42 from VZV and SVV under the JTT model (1,000 bootstrap replicates). SVV evolutionary distance was scaled to ×0.01 for visualization clarity. VZV clades are color-coded; the SVV branch is highlighted in red.


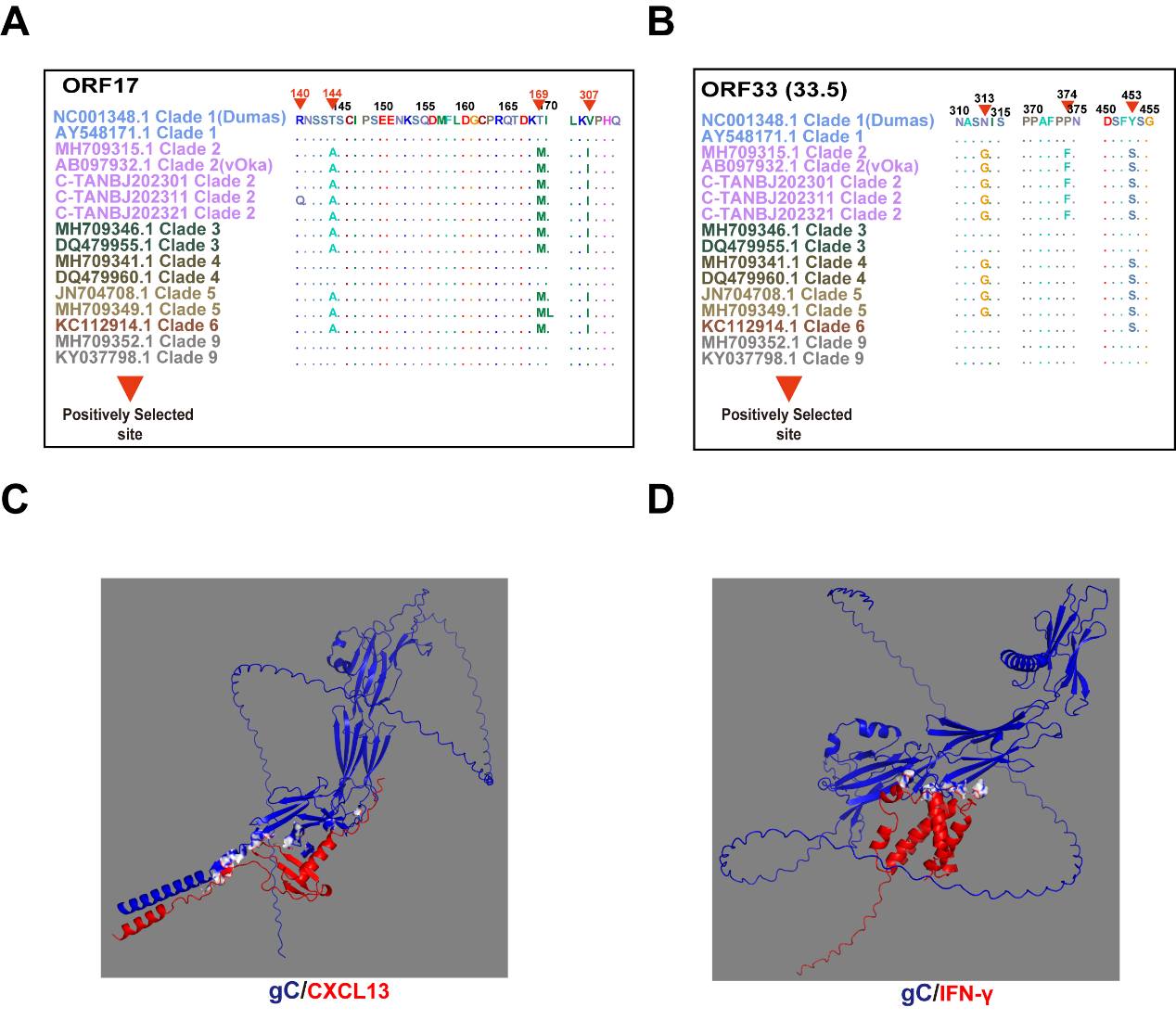


**Fig. S6 Positively selected sites in ORF17 and ORF33/ORF33.5 with computationally predicted gC binding to the CXCL13 and IFN-γ regions.** Schematic diagrams of positively selected sites in viral open reading frames (ORFs): ORF17 (**A**) and ORF33/ORF33.5 (**B**). AlphaFold-predicted protein interaction models between glycoprotein C (gC, blue) and immune ligands CXCL13 (red; **C**) or IFN-γ (red; **D**). Predicted binding regions highlighted in white.
