# Supplementary material for: Decoding VZV’s evolutionary arsenal: how Beijing strains use recombination and adaptive mutations to thrive
Source: Virus Evol. 2025 Sep 23;11(1):veaf076. doi: 10.1093/ve/veaf076 (PMC12513170; doi:10.1093/ve/veaf076)
Supplement: Supplementarytable_veaf076 [file supplementarytable_veaf076.docx]

**Table S1. Clinical Characteristics and WGS Metadata of Viral Strains.**

| **Sample ID** | **The assigned accession listed as follows** | **Age** | **Gender** | **Disease** | **Vaccine** | **Date of collect sample** | **Ct** | **10× coverage (%)** | **Depth** |
| --- | --- | --- | --- | --- | --- | --- | --- | --- | --- |
| BJ202313 | C_AA107092.1 | 2 years | female | Chickenpox | No | 23.11.21 | 18.2 | 99.99 | 7133.49 |
| BJ202314 | C_AA107093.1 | 6 months | female | Chickenpox | No | 23.11.21 | 22.44 | 99.63 | 412.81 |
| BJ202315 | C_AA107094.1 | 11 months | female | Chickenpox | No | 23.04.06 | 21.95 | 99.79 | 449.84 |
| BJ202316 | C_AA107095.1 | 5 years | male | Chickenpox | No | 23.05.31 | 20.53 | 97.94 | 40.54 |
| BJ202317 | N/A | 4 years | female | Chickenpox | No | 23.06.29 | 27.28 | 1.65 | 3.71 |
| BJ202318 | C_AA107096.1 | 1 years | female | Chickenpox | No | 23.06.29 | 18.91 | 99.42 | 113.15 |
| BJ202319 | C_AA107097.1 | 33 years | male | Herpes | No | 23.07.03 | 19.19 | 99.19 | 123.44 |
| BJ202320 | N/A | 10 days | male | Chickenpox | No | 23.09.06 | 32.74 | 0.05 | 2.61 |
| BJ202321 | C_AA107098.1 | 1 years | female | Chickenpox | No | 23.09.14 | 28.1 | 98.97 | 119.49 |
| BJ202322 | C_AA107099.1 | 6 years | male | Chickenpox | No | 23.11.12 | 19.15 | 99.31 | 225.84 |
| BJ202323 | C_AA107100.1 | 11 years | male | Chickenpox | No | 23.12.22 | 25.78 | 99.26 | 119.30 |
| BJ202324 | C_AA107101.1 | 3 months | male | Chickenpox | No | 23.12.24 | 26.11 | 98.84 | 78.60 |
| BJ202403 | C_AA107104.1 | 30 years | female | Herpes | No | 24.01.06 | 30.02 | 99.74 | 281.03 |
| BJ202404 | N/A | 4 years | male | Chickenpox | No | 23.06.11 | 32.52 | 15.01 | 7.20 |
| BJ202301 | C_AA107080.1 | 1 years | male | Chickenpox | No | 23.04.04 | 19.83 | 99.83 | 1260.70 |
| BJ202302 | C_AA107081.1 | 1 years | male | Chickenpox | No | 23.04.21 | 23.64 | 99.99 | 2880.06 |
| BJ202303 | C_AA107082.1 | 36 years | male | Herpes | No | 23.05.31 | 22.19 | 99.99 | 4766.81 |
| BJ202304 | C_AA107083.1 | 4 years | male | Chickenpox | No | 23.06.11 | 31.04 | 99.98 | 4693.44 |
| BJ202305 | C_AA107084.1 | 11 years | female | Chickenpox | No | 23.06.29 | 22.66 | 99.99 | 4752.14 |
| BJ202306 | C_AA107085.1 | 4 months | male | Chickenpox | No | 23.07.16 | 21.6 | 99.99 | 9037.59 |
| BJ202307 | C_AA107086.1 | 1 years | female | Chickenpox | No | 23.07.24 | 23.2 | 99.98 | 5622.45 |
| BJ202308 | C_AA107087.1 | 6 years | male | Chickenpox | No | 23.11.17 | 18.39 | 99.99 | 1686.35 |
| BJ202309 | C_AA107088.1 | 6 months | female | Chickenpox | No | 23.11.22 | 23.9 | 99.99 | 9629.24 |
| BJ202310 | C_AA107089.1 | 12 months | male | Chickenpox | No | 23.12.03 | 20.13 | 99.95 | 13172.2 |
| BJ202311 | C_AA107090.1 | 60 years | female | Herpes | No | 23.12.17 | 17.33 | 99.88 | 4640.27 |
| BJ202312 | C_AA107091.1 | 6 years | male | Chickenpox | No | 23.12.28 | 21.89 | 99.95 | 6687.29 |
| BJ202401 | C_AA107102.1 | 14 years | female | Chickenpox | No | 24.01.25 | 15.49 | 99.99 | 8479.43 |
| BJ202402 | C_AA107103.1 | 8 months | male | Chickenpox | No | 24.02.21 | 20.19 | 99.99 | 8557.76 |

N/A-no data available.

**Table S2. The proportion of characteristic variants of Clade 2 in each subclade.**

| **SNP** | **Clade2a** | **Clade2b.1** | **Clade2b.2** | **Clade2b.3** | **Clade2b.4** |
| --- | --- | --- | --- | --- | --- |
| C18082T (ORF12 tegument protein VP11/12) | 0.67 | 0.38 | 0.83 | 0 | 0.03 |
| A20711T (ORF14 envelope glycoprotein C) | 0.67 | 0.15 | 0.91 | 0.85 | 0.07 |
| T20753A (ORF14 envelope glycoprotein C) | 0.67 | 0.69 | 0.91 | 0.46 | 0.79 |
| A20795T (ORF14 envelope glycoprotein C) | 0.33 | 0.23 | 0 | 0.08 | 0.97 |
| T20837A (ORF14 envelope glycoprotein C) | 0.67 | 0.62 | 0.52 | 0.23 | 0.86 |
| A36926G (ORF22 large tegument protein) | 0 | 0 | 0 | 0.85 | 0 |
| C40130T (ORF22 large tegument protein) | 0 | 0 | 0 | 1 | 0.10 |
| A40370G (ORF22 large tegument protein) | 0 | 0 | 0 | 1 | 0.10 |
| G41452A (ORF22 large tegument protein) | 0 | 0.69 | 0.91 | 1 | 0.90 |
| A41499C (ORF22 large tegument protein) | 0.33 | 0.31 | 0.65 | 0.15 | 0.93 |
| T44995C (ORF26 DNA packaging protein UL32) | 0 | 0 | 0 | 1 | 0.10 |
| T54556C (between ORF29 and ORF30) | 1 | 0.54 | 0.04 | 0 | 0.03 |
| A60279C (between ORF29 and ORF30) | 0.67 | 0.85 | 0.04 | 0.31 | 0.31 |
| G76131T (ORF41 capsid triplex subunit 2) | 0 | 0 | 0 | 1 | 0.10 |
| C88867T (ORF51 DNA replication origin-binding helicase) | 0 | 0 | 0 | 1 | 0.14 |
| T99227A (ORF56 nuclear protein UL4) | 1 | 0.54 | 0.04 | 0.08 | 0.03 |
| C100653T (ORF59 uracil-DNA glycosylase) | 0 | 0 | 0 | 1 | 0.24 |
| G102192A (between ORF60 and ORF61) | 1 | 1 | 1 | 0.92 | 0.17 |
| A102203G (between ORF60 and ORF61) | 1 | 1 | 1 | 0.92 | 0.17 |
| C105010G (inverted repeat flanking UL) | 1 | 0.54 | 0.22 | 0.23 | 0.69 |
| T105145C (ORF62 transcriptional regulator ICP4) | 1 | 0.77 | 0.04 | 0 | 0 |
| T105146A (ORF62 transcriptional regulator ICP4) | 1 | 0.77 | 0.04 | 0 | 0 |
| T106262C (ORF62 transcriptional regulator ICP4) | 0 | 0 | 0.83 | 0.31 | 0 |
| C107607A (ORF62 transcriptional regulator ICP4) | 1 | 1 | 0.96 | 0.77 | 1 |
| G110050A (inverted repeat flanking US) | 1 | 0.54 | 0.04 | 0.08 | 0.03 |
| T110220G (oriS palindrome) | 0.33 | 0.31 | 0.78 | 0.38 | 0.76 |
| T110224G (oriS palindrome) | 0 | 0.54 | 0.91 | 0.54 | 0.86 |
| T110226G (oriS palindrome) | 0 | 0.54 | 0.87 | 0.54 | 0.90 |
| A110232G (oriS palindrome) | 1 | 1 | 0.78 | 0.92 | 0.90 |
| A110235G (oriS palindrome) | 1 | 0.85 | 0.39 | 0.15 | 0.07 |
| C110370A (inverted repeat flanking US) | 1 | 0.23 | 0 | 0.15 | 0.10 |
| G119527T (inverted repeat flanking US) | 1 | 0.23 | 0 | 0.08 | 0.14 |
| T119662C (oriS palindrome) | 1 | 0.77 | 0.26 | 0.69 | 0.14 |
| T119665C (oriS palindrome) | 1 | 0.92 | 1 | 0.77 | 0.97 |
| A119671C (oriS palindrome) | 0.67 | 0.31 | 0.96 | 0.62 | 0.90 |
| A119673C (oriS palindrome) | 0 | 0 | 0.91 | 0.54 | 0.83 |
| A119675C (oriS palindrome) | 0 | 0 | 0.96 | 0.54 | 0.83 |
| G122290T (ORF62 transcriptional regulator ICP4) | 1 | 1 | 0.96 | 0.77 | 1 |
| A123635G (ORF62 transcriptional regulator ICP4) | 0 | 0 | 0.83 | 0.31 | 0 |

**Table S3. The inter- and intra-clade recombinant events of the VZV.**

| **Group** | **sample_id** | **lineage_X** | **lineage_Y** | **mutation_pattern** | **raw_p_value** | **adjusted_p_value** | **X_mutations** | **Y_mutations** |
| --- | --- | --- | --- | --- | --- | --- | --- | --- |
| 1 | KY037798.1 Clade 9 | Clade 2 | Clade 3 | XXXXXXYXYYYYYYYYYY | 0.000192 | 0.004412 | 100114_A/101886_C/102192_A/102309_A/102458_G/103043_C/110232_G | 110169_A/110325_A/110326_A/112962_C/113243_C/116467_C/117413_A/119571_T/119572_T/119654_T/119728_T |
| 2 | KC112914.1 Clade 6 | Clade 2 | Clade 3 | XXXXXXYYYYY | 9.28E-05 | 0.001855 | 10079_A/11890_G/11906_G/13173_G/13407_A/17834_T | 23429_G/28111_G/29831_T/30206_G/30486_G |
| 3 | MH709312.1 Clade 9 | Clade 2 | Clade 5 | XXXXXYYYYYYYYYYYYY | 2.85E-06 | 6.83E-05 | 101886_C/102192_A/102309_A/102458_G/103043_C | 105012_C/105015_C/110169_A/110243_A/110325_A/110326_A/112962_C/113243_C/116467_C/119504_G/119511_A/119654_T/119728_T |
|  | MH709352.1 Clade 9 | Clade 2 | Clade 5 | XXXXXYYYYYYYYYYYYYYYY | 1.03E-07 | 2.37E-06 | 101886_C/102192_A/102309_A/102458_G/103043_C | 105012_C/105015_C/110169_A/110243_A/110325_A/110326_A/112962_C/113243_C/116467_C/119504_G/119511_A/119571_T/119572_T/119654_T/119665_C/119728_T |
|  | OQ454913.1 Clade 9 | Clade 2 | Clade 5 | XXXXXYYYYYYYYYYYYYYYYY | 3.53E-05 | 0.000812 | 101886_C/102192_A/102309_A/102458_G/103043_C | 105012_C/105015_C/110169_A/110243_A/110325_A/110326_A/112962_C/113243_C/116467_C/119504_G/119511_A/119519_G/119571_T/119572_T/119654_T/119665_C/119728_T |
|  | OQ835716.1 Clade 9 | Clade 2 | Clade 5 | XXXXXYYYYYYYYYYYYYYYY | 1.03E-07 | 2.37E-06 | 101886_C/102192_A/102309_A/102458_G/103043_C | 105012_C/105015_C/110169_A/110325_A/110326_A/112962_C/113243_C/116467_C/119504_G/119511_A/119519_G/119571_T/119572_T/119654_T/119665_C/119728_T |
| 4 | MH709313.1 | Clade 2b3 | Clade 2b4 | XXYYYY | 3.89E-12 | 5.84E-11 | 102192_A/102203_G | 107607_A/119665_C/119671_C/122290_T |
|  | PP261331.1 | Clade 2b3 | Clade 2b4 | XXYYYYYYYY | 2.23E-18 | 3.79E-17 | 102192_A/102203_G | 107607_A/110222_G/110224_G/110226_G/119665_C/119671_C/122290_T/124882_G |
| 5 | PP261331.1 | Clade 2b1 | Clade 2b4 | XXYYYX | 8.04E-24 | 1.37E-22 | 102192_A/102203_G/110235_G | 110222_G/110224_G/110226_G |
|  | KC112914.1 Clade 6 | Clade 2 | Clade 3 | YXXXXXXXYYYYYYYYX | 7.69E-08 | 2.00E-06 | 102309_A/102351_C/102458_G/102601_G/103043_C/105015_C/109654_T/120243_A | 102203_G/110169_A/110325_A/110326_A/112962_C/113243_C/119571_T/119572_T/119728_T |
|  | MH709312.1 Clade 9 | Clade 2 | Clade 3 | YXXXXXYYYYYYYYY | 7.58E-05 | 0.001365 | 102309_A/102458_G/103043_C/105012_C/105015_C | 102203_G/110169_A/110325_A/110326_A/112962_C/113243_C/116467_C/117413_A/119654_T/119728_T |
| 6 | KC112914.1 Clade 6 | Clade 5 | Clade 4 | YYYYYYYXXYXXXXXXXXXXXY | 1.31E-08 | 3.42E-07 | 105012_C/105015_C/110169_A/110232_G/110325_A/110326_A/112962_C/113243_C/119519_G/119571_T/119572_T/119665_C/119728_T | 99008_A/101886_C/102309_A/102351_C/102458_G/102601_G/103043_C/109654_T/120243_A |
|  | KP702725.1 Clade 6 | Clade 5 | Clade 4 | YYYYYYYXXYXXXXXXXXXXXY | 1.31E-08 | 3.42E-07 | 105012_C/105015_C/110169_A/110232_G/110325_A/110326_A/112962_C/113243_C/119519_G/119571_T/119572_T/119665_C/119728_T | 99008_A/101886_C/102309_A/102351_C/102458_G/102601_G/103043_C/109654_T/120243_A |
|  | MH709312.1 Clade 9 | Clade 5 | Clade 4 | YYYYYYYYXXXXXXXXXXXXX | 2.20E-07 | 5.29E-06 | 105012_C/105015_C/110169_A/110243_A/110325_A/110326_A/112962_C/113243_C/116467_C/119504_G/119511_A/119654_T/119728_T | 98437_C/98659_T/99008_A/101886_C/102192_A/102309_A/102458_G/103043_C |
|  | JN704710.1 Clade 9 | Clade 5 | Clade 4 | YYYYYYXXXXXX | 2.93E-06 | 4.68E-05 | 105012_C/105015_C/110169_A/112962_C/113243_C/116467_C | 97141_C/97780_A/98437_C/98659_T/102309_A/102458_G |
|  | KY037798.1 Clade 9 | Clade 5 | Clade 4 | YYYYYYYYYYXXXXXXXXXXXXXXXXX | 5.66E-06 | 0.00013 | 105015_C/110169_A/110232_G/110243_A/110325_A/110326_A/112962_C/113243_C/116467_C/119504_G/119511_A/119519_G/119571_T/119572_T/119654_T/119665_C/119728_T | 97141_C/97780_A/98437_C/98659_T/99008_A/101886_C/102192_A/102309_A/102458_G/103043_C |
| 7 | JN704708.1 Clade 5 | Clade 3 | Clade 4 | YYYYYYYYYYYYXXXXX | 0.000897 | 0.014358 | 110169_A/110325_A/110326_A/112962_C/113243_C | 89905_C/90217_C/90392_A/92026_G/93367_A/93368_A/94641_T/95241_C/95546_A/98825_C/100114_A/107307_C |
|  | KC112914.1 Clade 6 | Clade 3 | Clade 4 | YYYYYYYYYYYYXXXXX | 1.94E-07 | 3.89E-06 | 110169_A/110325_A/110326_A/112962_C/113243_C | 98437_C/98659_T/98825_C/99008_A/100114_A/101886_C/102309_A/102351_C/102458_G/102601_G/103043_C/109654_T |
|  | KP702725.1 Clade 6 | Clade 3 | Clade 4 | YYYYYYYYYYYYXXXXX | 1.94E-07 | 3.89E-06 | 110169_A/110325_A/110326_A/112962_C/113243_C | 98437_C/98659_T/98825_C/99008_A/100114_A/101886_C/102309_A/102351_C/102458_G/102601_G/103043_C/109654_T |
|  | KY037797.1 Clade 5 | Clade 3 | Clade 4 | YYYYYYYYYYYYXXXXX | 0.00241 | 0.038564 | 110169_A/110325_A/110326_A/112962_C/113243_C | 89905_C/90217_C/90392_A/92026_G/93367_A/93368_A/94641_T/95241_C/95546_A/98825_C/100114_A/107307_C |
|  | MH709351.1 Clade 5 | Clade 3 | Clade 4 | YYYYYYYYYYXXXXX | 0.002604 | 0.041663 | 110169_A/110325_A/110326_A/112962_C/113243_C | 92026_G/93367_A/93368_A/94641_T/95241_C/95546_A/98825_C/100114_A/102192_A/107307_C |
|  | MH709358.1 Clade 5 | Clade 3 | Clade 4 | YYYYYYYYYYYYXXXXX | 0.00241 | 0.038564 | 110169_A/110325_A/110326_A/112962_C/113243_C | 89905_C/90217_C/90392_A/92026_G/93367_A/93368_A/94641_T/95241_C/95546_A/98825_C/100114_A/107307_C |
|  | OQ835716.1 Clade 9 | Clade 3 | Clade 4 | YYYYYYYYYYYYYYYYYYYYXXXXX | 2.91E-11 | 6.40E-10 | 110169_A/110325_A/110326_A/112962_C/113243_C | 90392_A/92026_G/92523_G/93367_A/93368_A/94641_T/95241_C/95546_A/97141_C/97780_A/98437_C/98659_T/98825_C/99008_A/100114_A/101886_C/102192_A/102309_A/102458_G/103043_C |
|  | MH709352.1 Clade 9 | Clade 3 | Clade 4 | YYYYYYYYYYYYYYYXXXXXXX | 1.40E-09 | 3.35E-08 | 110169_A/110325_A/110326_A/112962_C/113243_C/116467_C/117413_A | 94641_T/95241_C/95546_A/97141_C/97780_A/98437_C/98659_T/98825_C/99008_A/100114_A/101886_C/102192_A/102309_A/102458_G/103043_C |
|  | OQ454913.1 Clade 9 | Clade 3 | Clade 4 | YYYYYYYYYYYYYYYXXXXXXX | 1.34E-07 | 3.36E-06 | 110169_A/110325_A/110326_A/112962_C/113243_C/116467_C/117413_A | 94641_T/95241_C/95546_A/97141_C/97780_A/98437_C/98659_T/98825_C/99008_A/100114_A/101886_C/102192_A/102309_A/102458_G/103043_C |
|  | KY037798.1 Clade 9 | Clade 3 | Clade 4 | YYYYYYYYYYYYYYXXXXXXXXXXX | 4.99E-07 | 1.30E-05 | 110169_A/110325_A/110326_A/112962_C/113243_C/116467_C/117413_A/119571_T/119572_T/119654_T/119728_T | 95241_C/95546_A/97141_C/97780_A/98437_C/98659_T/98825_C/99008_A/100114_A/101886_C/102192_A/102309_A/102458_G/103043_C |
|  | MH709312.1 Clade 9 | Clade 3 | Clade 4 | YYYYYYYXXXXXXXXX | 1.85E-06 | 4.44E-05 | 110169_A/110325_A/110326_A/112962_C/113243_C/116467_C/117413_A/119654_T/119728_T | 99008_A/100114_A/101886_C/102192_A/102309_A/102458_G/103043_C |
|  | JN704706.1 Clade 5 | Clade 3 | Clade 4 | YYYYYXXXXXXXXXX | 0.003264 | 0.045695 | 110169_A/110325_A/110326_A/112962_C/113243_C/116467_C/119571_T/119572_T/119654_T/119728_T | 95241_C/95546_A/98825_C/100114_A/107307_C |
|  | JN704710.1 Clade 9 | Clade 3 | Clade 4 | YYYYYYYYYYYYXXXXX | 1.07E-06 | 2.47E-05 | 110169_A/112962_C/113243_C/116467_C/117413_A | 93367_A/93368_A/94641_T/95241_C/97141_C/97780_A/98437_C/98659_T/98825_C/100114_A/102309_A/102458_G |
| 8 | KJ767491.1 | Clade 2b1 | Clade 2b2 | YYYXXYYY | 1.76E-16 | 2.47E-15 | 110232_G/110235_G | 110222_G/110224_G/110226_G/119671_C/119673_C/119675_C |
|  | KU926315.1 | Clade 2b1 | Clade 2b2 | YYXXY | 4.26E-18 | 6.81E-17 | 110232_G/110235_G | 110224_G/110226_G/119671_C |
|  | KU926316.1 | Clade 2b1 | Clade 2b2 | YYYYXXYYYY | 4.40E-24 | 7.04E-23 | 110232_G/110235_G | 106262_C/110222_G/110224_G/110226_G/119671_C/119673_C/119675_C/123635_G |
|  | KU926317.1 | Clade 2b1 | Clade 2b2 | YYYYXXYYYY | 3.47E-21 | 5.55E-20 | 110232_G/110235_G | 106262_C/110222_G/110224_G/110226_G/119671_C/119673_C/119675_C/123635_G |
| 9 | PP261331.1 | Clade 2a | Clade 2b4 | YYYXXXXYY | 4.22E-19 | 5.91E-18 | 110235_G/110370_A/119527_T/119662_C | 110222_G/110224_G/110226_G/119671_C/124882_G |
| 10 | KP702725.1 Clade 6 | Clade 2 | Clade 3 | XXXXXYYYYYYYYY | 6.75E-06 | 0.000135 | 11890_G/11906_G/13173_G/13407_A/17834_T | 23429_G/28111_G/29831_T/30206_G/30486_G/32255_T/33068_C/33473_T/35141_T |
| 11 | PP261331.1 | Clade 2a | Clade 2b4 | XXYY | 1.86E-11 | 2.23E-10 | 119527_T/119662_C | 119671_C/124882_G |
| 12 | JN704705.1 Clade 5 | Clade 3 | Clade 4 | YYYYYYYYYYYXXXXXXXXX | 0.000845 | 0.016909 | 14197_A/17365_C/17725_G/18054_C/23294_G/23429_G/24092_C/24578_G/24654_T | 789_C/790_C/791_C/3764_G/6652_A/6850_A/6853_T/7091_A/11890_G/11906_G/12188_A |
|  | JN704708.1 Clade 5 | Clade 3 | Clade 4 | YYYYYYYYYYYXXXXXXXXX | 0.000845 | 0.016909 | 14197_A/17365_C/17725_G/18054_C/23294_G/23429_G/24092_C/24578_G/24654_T | 789_C/790_C/791_C/3764_G/6652_A/6850_A/6853_T/7091_A/11890_G/11906_G/12188_A |
| 13 | JN704701.1 Clade 3 | Clade 3 | Clade 5 | YYYYYXXXXX | 6.99E-06 | 9.79E-05 | 15896_T/20656_A/20684_C/21734_T/35141_T | 13977_A/14042_C/14090_C/14194_G/14195_A |
|  | MH709317.1 Clade 3 | Clade 3 | Clade 5 | YYYYYXXXXX | 6.99E-06 | 9.79E-05 | 15896_T/20656_A/20684_C/21734_T/35141_T | 13977_A/14042_C/14090_C/14194_G/14195_A |
| 14 | MH709339.1 | Clade 2b2 | Clade 5 | XXXXXXYYXXXXXXXXXXX | 4.03E-05 | 0.000604 | 17834_T/19719_G/20656_A/20684_C/20711_T/20753_A/21371_A/21734_T/22504_G/22794_G/32274_G/37649_G/37902_G/38055_C/38177_A/38717_T/39263_A | 20837_A/20963_T |
| 15 | MH709313.1 | Clade 2b2 | Clade 2b4 | XXYY | 7.95E-14 | 1.59E-12 | 20711_T/20753_A | 20795_T/20837_A |
|  | MH709339.1 | Clade 2b2 | Clade 2b4 | XXYY | 1.29E-16 | 2.06E-15 | 20711_T/20753_A | 20837_A/41499_C |
| 16 | KC112914.1 Clade 6 | Clade 3 | Clade 4 | YYYYYYXXXXXXXXXXXX | 2.43E-06 | 4.87E-05 | 23294_G/23429_G/24578_G/24654_T/25067_A/28111_G/29831_T/30206_G/30486_G/32255_T/33068_C/33473_T | 10079_A/11890_G/11906_G/13173_G/13407_A/17834_T |
| 17 | JN704709.1 CladeⅧ | Clade 3 | Clade 4 | XXXXXXXXYYXXXXXXXXYYYYY | 6.73E-06 | 0.000121 | 28111_G/29831_T/30206_G/30486_G/32255_T/33068_C/33473_T/35141_T/38714_T/39394_A/39530_G/41618_T/42069_G/42476_G/43262_C/44835_T | 38081_C/38177_A/48050_C/51200_T/51840_A/52917_G/52977_G |
| 18 | KP702725.1 Clade 6 | Clade 3 | Clade 4 | XXXYYXXXXXXXXYYYYY | 4.56E-07 | 6.84E-06 | 33068_C/33473_T/35141_T/38714_T/39394_A/39530_G/41618_T/42069_G/42476_G/43262_C/44835_T | 38081_C/38177_A/48050_C/52917_G/52977_G/57301_C/57955_T |
|  | JN704708.1 Clade 5 | Clade 3 | Clade 4 | XXYXXXXXXXYYYYYY | 2.02E-05 | 0.000303 | 33068_C/33473_T/38714_T/39394_A/39530_G/41618_T/42069_G/42476_G/43262_C | 38081_C/48050_C/52365_T/52917_G/52977_G/57301_C/57955_T |
|  | JN704705.1 Clade 5 | Clade 3 | Clade 4 | XXYXXXXXXXXYYYYYY | 3.95E-05 | 0.000593 | 33068_C/33473_T/38714_T/39394_A/39530_G/41618_T/42069_G/42476_G/43262_C/44835_T | 38081_C/48050_C/52365_T/52917_G/52977_G/57301_C/57955_T |
|  | MH709340.1 Clade 5 | Clade 3 | Clade 4 | XXYXXXXXXXXYYYYYY | 0.000533 | 0.007993 | 33068_C/33473_T/38714_T/39394_A/39530_G/41618_T/42069_G/42476_G/43262_C/44835_T | 38081_C/48050_C/52365_T/52917_G/52977_G/57301_C/57955_T |
|  | MH709342.1 Clade 5 | Clade 3 | Clade 4 | XXYXXXXXXXXYYYYYY | 0.001572 | 0.023577 | 33068_C/33473_T/38714_T/39394_A/39530_G/41618_T/42069_G/42476_G/43262_C/44835_T | 38081_C/48050_C/52365_T/52917_G/52977_G/57301_C/57955_T |
|  | MH709349.1 Clade 5 | Clade 3 | Clade 4 | XXYXXXXXXXXYYYYYY | 0.000533 | 0.007993 | 33068_C/33473_T/38714_T/39394_A/39530_G/41618_T/42069_G/42476_G/43262_C/44835_T | 38081_C/48050_C/52365_T/52917_G/52977_G/57301_C/57955_T |
|  | MH709351.1 Clade 5 | Clade 3 | Clade 4 | XXYXXXXXXXXYYYYYY | 0.000158 | 0.002363 | 33068_C/33473_T/38714_T/39394_A/39530_G/41618_T/42069_G/42476_G/43262_C/44835_T | 38081_C/48050_C/52365_T/52917_G/52977_G/57301_C/57955_T |
|  | MH709358.1 Clade 5 | Clade 3 | Clade 4 | XXYXXXXXXXXYYYYYY | 0.000158 | 0.002363 | 33068_C/33473_T/38714_T/39394_A/39530_G/41618_T/42069_G/42476_G/43262_C/44835_T | 38081_C/48050_C/52365_T/52917_G/52977_G/57301_C/57955_T |
|  | MH709360.1 Clade 5 | Clade 3 | Clade 4 | XXYXXXXXXXXYYYYYY | 0.001572 | 0.023577 | 33068_C/33473_T/38714_T/39394_A/39530_G/41618_T/42069_G/42476_G/43262_C/44835_T | 38081_C/48050_C/52365_T/52917_G/52977_G/57301_C/57955_T |
|  | JN704704.1 Clade 5 | Clade 3 | Clade 4 | XXYXXXXXXXYYYYY | 0.000731 | 0.010966 | 33068_C/33473_T/38714_T/39394_A/39530_G/42069_G/42476_G/43262_C/44835_T | 38081_C/52365_T/52917_G/52977_G/57301_C/57955_T |
| 19 | KC112914.1 Clade 6 | Clade 2 | Clade 3 | YYYYYYYYXXXXXX | 4.59E-05 | 0.000735 | 38081_C/38177_A/41452_A/41499_C/48050_C/52917_G | 28111_G/29831_T/30206_G/30486_G/32255_T/33068_C/33473_T/35141_T |
| 20 | KC112914.1 Clade 6 | Clade 3 | Clade 4 | YYXXXXXXXXYYYYYYY | 4.96E-06 | 7.44E-05 | 38714_T/39394_A/39530_G/41618_T/42069_G/42476_G/43262_C/44835_T | 38081_C/38177_A/48050_C/52917_G/52977_G/57301_C/57955_T/60405_A/60781_G |
|  | KY037797.1 Clade 5 | Clade 3 | Clade 4 | YXXXXXXXXYYYYYYYYYY | 0.001965 | 0.02947 | 38714_T/39394_A/39530_G/41618_T/42069_G/42476_G/43262_C/44835_T | 38081_C/48050_C/52365_T/52917_G/52977_G/57301_C/57955_T/60405_A/60781_G/61201_C/61202_C |
|  | MH499466.1 Clade 5 | Clade 3 | Clade 4 | YXXXXXXXXYYYYYYYYYY | 0.001965 | 0.02947 | 38714_T/39394_A/39530_G/41618_T/42069_G/42476_G/43262_C/44835_T | 38081_C/48050_C/52365_T/52917_G/52977_G/57301_C/57955_T/60405_A/60781_G/61201_C/61202_C |
|  | MH499469.1 Clade 5 | Clade 3 | Clade 4 | YXXXXXXXXYYYYYYYYYY | 0.001965 | 0.02947 | 38714_T/39394_A/39530_G/41618_T/42069_G/42476_G/43262_C/44835_T | 38081_C/48050_C/52365_T/52917_G/52977_G/57301_C/57955_T/60405_A/60781_G/61201_C/61202_C |
|  | MH709321.1 Clade 5 | Clade 3 | Clade 4 | YXXXXXXXXYYYYYYYYYY | 0.00024 | 0.003593 | 38714_T/39394_A/39530_G/41618_T/42069_G/42476_G/43262_C/44835_T | 38081_C/48050_C/52365_T/52917_G/52977_G/57301_C/57955_T/60405_A/60781_G/61201_C/61202_C |
|  | MH709324.1 Clade 5 | Clade 3 | Clade 4 | YYXXXXXXXXYYYYYYYYYY | 0.000852 | 0.012778 | 38714_T/39394_A/39530_G/41618_T/42069_G/42476_G/43262_C/44835_T | 38055_C/38081_C/48050_C/52365_T/52917_G/52977_G/57301_C/57955_T/60405_A/60781_G/61201_C/61202_C |
|  | MH709325.1 Clade 5 | Clade 3 | Clade 4 | YXXXXXXXXYYYYYYYYYY | 0.000726 | 0.010893 | 38714_T/39394_A/39530_G/41618_T/42069_G/42476_G/43262_C/44835_T | 38081_C/48050_C/52365_T/52917_G/52977_G/57301_C/57955_T/60405_A/60781_G/61201_C/61202_C |
|  | MH709330.1 Clade 5 | Clade 3 | Clade 4 | YXXXXXXXXYYYYYYYYYY | 0.001965 | 0.02947 | 38714_T/39394_A/39530_G/41618_T/42069_G/42476_G/43262_C/44835_T | 38081_C/48050_C/52365_T/52917_G/52977_G/57301_C/57955_T/60405_A/60781_G/61201_C/61202_C |
|  | MH709332.1 Clade 5 | Clade 3 | Clade 4 | YXXXXXXXXYYYYYYYYYY | 0.000726 | 0.010893 | 38714_T/39394_A/39530_G/41618_T/42069_G/42476_G/43262_C/44835_T | 38081_C/48050_C/52365_T/52917_G/52977_G/57301_C/57955_T/60405_A/60781_G/61201_C/61202_C |
|  | MH709343.1 Clade 5 | Clade 3 | Clade 4 | YXXXXXXXXYYYYYYYYYY | 0.002134 | 0.032007 | 38714_T/39394_A/39530_G/41618_T/42069_G/42476_G/43262_C/44835_T | 38081_C/48050_C/52365_T/52917_G/52977_G/57301_C/57955_T/60405_A/60781_G/61201_C/61202_C |
|  | MH709345.1 Clade 5 | Clade 3 | Clade 4 | YXXXXXXXXYYYYYYYYYY | 0.002134 | 0.032007 | 38714_T/39394_A/39530_G/41618_T/42069_G/42476_G/43262_C/44835_T | 38081_C/48050_C/52365_T/52917_G/52977_G/57301_C/57955_T/60405_A/60781_G/61201_C/61202_C |
|  | MH709373.1 Clade 5 | Clade 3 | Clade 4 | YXXXXXXXXYYYYYYYYYY | 0.002134 | 0.032007 | 38714_T/39394_A/39530_G/41618_T/42069_G/42476_G/43262_C/44835_T | 38081_C/48050_C/52365_T/52917_G/52977_G/57301_C/57955_T/60405_A/60781_G/61201_C/61202_C |
|  | OQ427944.1 Clade 5 | Clade 3 | Clade 4 | YXXXXXXXXYYYYYYYYYY | 0.002134 | 0.032007 | 38714_T/39394_A/39530_G/41618_T/42069_G/42476_G/43262_C/44835_T | 38081_C/48050_C/52365_T/52917_G/52977_G/57301_C/57955_T/60405_A/60781_G/61201_C/61202_C |
|  | OQ723678.1 Clade 5 | Clade 3 | Clade 4 | YXXXXXXXXYYYYYYYYYY | 0.002134 | 0.032007 | 38714_T/39394_A/39530_G/41618_T/42069_G/42476_G/43262_C/44835_T | 38081_C/48050_C/52365_T/52917_G/52977_G/57301_C/57955_T/60405_A/60781_G/61201_C/61202_C |
|  | OQ916050.1 Clade 5 | Clade 3 | Clade 4 | YXXXXXXXXYYYYYYYYYY | 0.002134 | 0.032007 | 38714_T/39394_A/39530_G/41618_T/42069_G/42476_G/43262_C/44835_T | 38081_C/48050_C/52365_T/52917_G/52977_G/57301_C/57955_T/60405_A/60781_G/61201_C/61202_C |
|  | PP169944.1 Clade 5 | Clade 3 | Clade 4 | YXXXXXXXXYYYYYYYYYY | 0.002134 | 0.032007 | 38714_T/39394_A/39530_G/41618_T/42069_G/42476_G/43262_C/44835_T | 38081_C/48050_C/52365_T/52917_G/52977_G/57301_C/57955_T/60405_A/60781_G/61201_C/61202_C |
|  | JN704704.1 Clade 5 | Clade 2 | Clade 4 | YXXXXXXXYYYYYYY | 0.001014 | 0.015214 | 38714_T/39394_A/39530_G/42069_G/42476_G/43262_C/44835_T | 38081_C/52365_T/52917_G/52977_G/57301_C/57955_T/60405_A/60781_G |
|  | JN704706.1 Clade 5 | Clade 2 | Clade 4 | YXXXXXXXYYYYYYYYYY | 1.71E-05 | 0.000256 | 38714_T/39394_A/39530_G/42069_G/42476_G/43262_C/44835_T | 38081_C/48050_C/52365_T/52917_G/52977_G/57301_C/57955_T/60405_A/60781_G/61201_C/61202_C |
|  | JN704707.1 Clade 5 | Clade 3 | Clade 4 | YXXXXXXXYYYYYYYYYY | 0.001771 | 0.026558 | 38714_T/39394_A/41618_T/42069_G/42476_G/43262_C/44835_T | 38081_C/48050_C/52365_T/52917_G/52977_G/57301_C/57955_T/60405_A/60781_G/61201_C/61202_C |
| 21 | C-TAN/BJ202310 | Clade 2b3 | Clade 2b4 | YYXXY | 3.06E-15 | 6.43E-14 | 40130_T/40370_G | 20795_T/20837_A/41499_C |
|  | C-TAN/BJ202321 | Clade 2b3 | Clade 2b4 | YYXXY | 1.46E-16 | 2.34E-15 | 40130_T/40370_G | 20795_T/20837_A/41499_C |
|  | C-TAN/BJ202401 | Clade 2b3 | Clade 2b4 | YYXXY | 3.06E-15 | 6.43E-14 | 40130_T/40370_G | 20795_T/20837_A/41499_C |
| 22 | MH709328.1 Clade 5 | Clade 3 | Clade 4 | XXXXXYYYYYYYYYYYYY | 0.000594 | 0.011886 | 41618_T/42069_G/42476_G/43262_C/44835_T | 48050_C/52365_T/52917_G/52977_G/57301_C/57955_T/60405_A/60781_G/61201_C/61202_C/64375_C/64703_A/64740_T |
|  | MH709365.1 Clade 5 | Clade 3 | Clade 4 | XXXXXYYYYYYYYYYYYY | 0.001394 | 0.02789 | 41618_T/42069_G/42476_G/43262_C/44835_T | 48050_C/52365_T/52917_G/52977_G/57301_C/57955_T/60405_A/60781_G/61201_C/61202_C/64375_C/64703_A/64740_T |
|  | MH709374.1 Clade 5 | Clade 3 | Clade 4 | XXXXXYYYYYYYYYYYYY | 0.001394 | 0.02789 | 41618_T/42069_G/42476_G/43262_C/44835_T | 48050_C/52365_T/52917_G/52977_G/57301_C/57955_T/60405_A/60781_G/61201_C/61202_C/64375_C/64703_A/64740_T |
|  | KC112914.1 Clade 6 | Clade 3 | Clade 4 | XXXXXYYYYYYYXY | 4.69E-06 | 6.56E-05 | 41618_T/42069_G/42476_G/43262_C/44835_T/64191_A | 48050_C/52917_G/52977_G/57301_C/57955_T/60405_A/60781_G/64375_C |
|  | KP702725.1 Clade 6 | Clade 3 | Clade 4 | XXXXXYYYYYYYXY | 2.33E-05 | 0.000327 | 41618_T/42069_G/42476_G/43262_C/44835_T/64191_A | 48050_C/52917_G/52977_G/57301_C/57955_T/60405_A/60781_G/64375_C |
| 23 | C-TAN/BJ202310 | Clade 2b3 | Clade 2b4 | XXYYYY | 8.52E-23 | 1.28E-21 | 44995_C/76131_T/88867_T/100653_T | 107607_A/110222_G/110224_G/110226_G |
|  | C-TAN/BJ202321 | Clade 2b3 | Clade 2b4 | XXYYYY | 1.58E-23 | 2.53E-22 | 44995_C/76131_T/88867_T/100653_T | 107607_A/110222_G/110224_G/110226_G |
|  | C-TAN/BJ202401 | Clade 2b3 | Clade 2b4 | XXYYYY | 8.52E-23 | 1.28E-21 | 44995_C/76131_T/88867_T/100653_T | 107607_A/110222_G/110224_G/110226_G |
| 24 | KC112914.1 Clade 6 | Clade 3 | Clade 4 | YYYYYYXYXXXXXXX | 1.10E-07 | 1.87E-06 | 64191_A/66112_G/68254_T/69424_A/70014_A/71196_T/75103_G/76779_A | 52917_G/52977_G/57301_C/57955_T/60405_A/60781_G/64375_C |
|  | KP702725.1 Clade 6 | Clade 3 | Clade 4 | YYYYYYXYXXXXXXX | 1.10E-07 | 1.87E-06 | 64191_A/66112_G/68254_T/69424_A/70014_A/71196_T/75103_G/76779_A | 52917_G/52977_G/57301_C/57955_T/60405_A/60781_G/64375_C |
|  | JN704709.1 CladeⅧ | Clade 3 | Clade 4 | YYYYYYYYXYXXXXX | 2.13E-07 | 2.98E-06 | 64191_A/66112_G/68254_T/70014_A/71196_T/75103_G | 51200_T/51840_A/52917_G/52977_G/57301_C/57955_T/60405_A/60781_G/64375_C |
| 25 | JN704709.1 CladeⅧ | Clade 2 | Clade 3 | XXXXXXXXXYYYYY | 3.64E-07 | 8.00E-06 | 6853_T/7091_A/10079_A/11890_G/11906_G/13173_G/13407_A/17834_T/20837_A | 23429_G/28111_G/29831_T/30206_G/30486_G |
| 26 | OQ454913.1 Clade 9 | Clade 3 | Clade 4 | XXXXXXXXXXXXYYYYYY | 7.87E-05 | 0.001338 | 69424_A/70014_A/75103_G/76779_A/77154_C/78385_T/78545_A/80306_T/81854_A/82141_C/86620_A/88477_T | 89905_C/90392_A/92026_G/92523_G/93367_A/93368_A |
|  | OQ835716.1 Clade 9 | Clade 3 | Clade 4 | XXXXXXXXXXYYYYYYYYYYYYYYY | 2.33E-10 | 6.06E-09 | 75103_G/76779_A/77154_C/78385_T/78545_A/80306_T/81854_A/82141_C/86620_A/88477_T | 89905_C/90392_A/92026_G/92523_G/93367_A/93368_A/94641_T/95241_C/95546_A/97141_C/97780_A/98437_C/98659_T/98825_C/99008_A |
| 27 | JN704709.1 CladeⅧ | Clade 3 | Clade 4 | XXXXXXXYYXXYYYYYY | 6.15E-06 | 8.61E-05 | 75103_G/76779_A/77154_C/78385_T/80306_T/81854_A/82141_C/86620_A/88477_T | 84983_A/85563_T/89905_C/90392_A/92026_G/92523_G/98437_C/98659_T |
|  | JN704710.1 Clade 9 | Clade 3 | Clade 4 | XXXXXXYYYYYYYYYYY | 6.55E-06 | 0.000131 | 76779_A/78545_A/80306_T/82141_C/86620_A/88477_T | 89905_C/93367_A/93368_A/94641_T/95241_C/97141_C/97780_A/98437_C/98659_T/98825_C/100114_A |
| 28 | MH709317.1 Clade 3 | Clade 3 | Clade 5 | XXXXXYYYYY | 5.14E-08 | 8.22E-07 | 77154_C/78385_T/78545_A/80306_T/81854_A | 89905_C/90392_A/92026_G/100114_A |
|  | MH709346.1 Clade 3 | Clade 3 | Clade 5 | XXXXXYYYYY | 5.14E-08 | 8.22E-07 | 77154_C/78385_T/78545_A/80306_T/81854_A | 89905_C/90392_A/92026_G/100114_A/101464_A |
|  | MH709375.1 Clade 3 | Clade 3 | Clade 5 | XXXXXYYYYY | 5.14E-08 | 8.22E-07 | 77154_C/78385_T/78545_A/80306_T/81854_A | 89905_C/90392_A/92026_G/100114_A |
| 29 | MH709339.1 | Clade 2b2 | Clade 5 | XXXXXYYXXXXXXXXXX | 0.000119 | 0.002267 | 7753_T/9460_C/10079_A/13173_G/13407_A/17834_T/19719_G/20656_A/20684_C/20711_T/20753_A/21371_A/21734_T/22504_G/22794_G | 14042_C/14197_A |
| 30 | KC112914.1 Clade 6 | Clade 3 | Clade 4 | XXXXXYYYYYYYYYYY | 2.28E-07 | 3.19E-06 | 80306_T/81854_A/82141_C/86620_A/88477_T | 98437_C/98659_T/98825_C/99008_A/100114_A/101886_C/102309_A/102351_C/102458_G/102601_G/103043_C |
|  | KY037798.1 Clade 9 | Clade 3 | Clade 4 | XXXXXYYYYYYYYYYYYYYYYYYYYY | 1.10E-11 | 1.54E-10 | 80306_T/81854_A/82141_C/86620_A/88477_T | 89905_C/90392_A/92026_G/92523_G/93367_A/93368_A/94641_T/95241_C/95546_A/97141_C/97780_A/98437_C/98659_T/98825_C/99008_A/100114_A/101886_C/102192_A/102309_A/102458_G/103043_C |
|  | MH709312.1 Clade 9 | Clade 3 | Clade 4 | XXXXXYYYYYYYYYYYYYYYYYYYYY | 3.74E-11 | 5.23E-10 | 80306_T/81854_A/82141_C/86620_A/88477_T | 89905_C/90392_A/92026_G/92523_G/93367_A/93368_A/94641_T/95241_C/95546_A/97141_C/97780_A/98437_C/98659_T/98825_C/99008_A/100114_A/101886_C/102192_A/102309_A/102458_G/103043_C |
|  | MH709352.1 Clade 9 | Clade 3 | Clade 4 | XXXXXYYYYYYYYYYYYYYYYYYYYY | 1.10E-11 | 1.54E-10 | 80306_T/81854_A/82141_C/86620_A/88477_T | 89905_C/90392_A/92026_G/92523_G/93367_A/93368_A/94641_T/95241_C/95546_A/97141_C/97780_A/98437_C/98659_T/98825_C/99008_A/100114_A/101886_C/102192_A/102309_A/102458_G/103043_C |
| 31 | MH709352.1 Clade 9 | Clade 5 | Clade 4 | XXYYYYYYYYYYYXXXXXXX | 3.46E-10 | 5.88E-09 | 86620_A/88477_T/105012_C/105015_C/110169_A/110232_G/110243_A/110325_A/110326_A | 92523_G/97141_C/97780_A/98437_C/98659_T/99008_A/101886_C/102192_A/102309_A/102458_G/103043_C |
|  | OQ454913.1 Clade 9 | Clade 5 | Clade 4 | XXYYYYYYYYYYYXXXXXXX | 3.11E-07 | 5.29E-06 | 86620_A/88477_T/105012_C/105015_C/110169_A/110232_G/110243_A/110325_A/110326_A | 92523_G/97141_C/97780_A/98437_C/98659_T/99008_A/101886_C/102192_A/102309_A/102458_G/103043_C |
|  | OQ835716.1 Clade 9 | Clade 5 | Clade 4 | XXYYYYYYYYYYYXXXXXX | 8.29E-10 | 1.41E-08 | 86620_A/88477_T/105012_C/105015_C/110169_A/110232_G/110325_A/110326_A | 92523_G/97141_C/97780_A/98437_C/98659_T/99008_A/101886_C/102192_A/102309_A/102458_G/103043_C |
|  | KY037798.1 Clade 9 | Clade 5 | Clade 4 | XXYYYYYYYYYYYXXXXXX | 6.59E-07 | 1.12E-05 | 86620_A/88477_T/105015_C/110169_A/110232_G/110243_A/110325_A/110326_A | 92523_G/97141_C/97780_A/98437_C/98659_T/99008_A/101886_C/102192_A/102309_A/102458_G/103043_C |
|  | MH709312.1 Clade 9 | Clade 5 | Clade 4 | XYYYYYYYYYYYXXXXXXX | 7.50E-10 | 1.35E-08 | 88477_T/105012_C/105015_C/110169_A/110243_A/110325_A/110326_A/112962_C | 92523_G/97141_C/97780_A/98437_C/98659_T/99008_A/101886_C/102192_A/102309_A/102458_G/103043_C |
| 32 | MH709317.1 Clade 3 | Clade 2 | Clade 3 | XXXXXYYYYYY | 9.78E-05 | 0.001369 | 92026_G/100114_A/105012_C/105015_C/110050_A/110232_G | 110169_A/110325_A/110326_A/112962_C/113243_C/116467_C |
|  | MH709346.1 Clade 3 | Clade 2 | Clade 3 | XXXXXYXYYYYY | 0.000371 | 0.005195 | 92026_G/100114_A/105012_C/105015_C/110050_A/110232_G | 110169_A/110325_A/110326_A/112962_C/113243_C/116467_C |
|  | MH709375.1 Clade 3 | Clade 2 | Clade 3 | XXXXXYYYYYY | 9.78E-05 | 0.001369 | 92026_G/100114_A/105012_C/105015_C/110050_A/110232_G | 110169_A/110325_A/110326_A/112962_C/113243_C/116467_C |

**Table S4.** **The correspondence between the sequence number in the GenBase and the number used in the study.**

| Number in the paper | Number in the GenBase |
| --- | --- |
| C-TAN/BJ202301 | C_AA107080.1 |
| C-TAN/BJ202302 | C_AA107081.1 |
| C-TAN/BJ202303 | C_AA107082.1 |
| C-TAN/BJ202304 | C_AA107083.1 |
| C-TAN/BJ202305 | C_AA107084.1 |
| C-TAN/BJ202306 | C_AA107085.1 |
| C-TAN/BJ202307 | C_AA107086.1 |
| C-TAN/BJ202308 | C_AA107087.1 |
| C-TAN/BJ202309 | C_AA107088.1 |
| C-TAN/BJ202310 | C_AA107089.1 |
| C-TAN/BJ202311 | C_AA107090.1 |
| C-TAN/BJ202312 | C_AA107091.1 |
| C-TAN/BJ202313 | C_AA107092.1 |
| C-TAN/BJ202314 | C_AA107093.1 |
| C-TAN/BJ202315 | C_AA107094.1 |
| C-TAN/BJ202316 | C_AA107095.1 |
| C-TAN/BJ202318 | C_AA107096.1 |
| C-TAN/BJ202319 | C_AA107097.1 |
| C-TAN/BJ202321 | C_AA107098.1 |
| C-TAN/BJ202322 | C_AA107099.1 |
| C-TAN/BJ202323 | C_AA107100.1 |
| C-TAN/BJ202324 | C_AA107101.1 |
| C-TAN/BJ202401 | C_AA107102.1 |
| C-TAN/BJ202402 | C_AA107103.1 |
| C-TAN/BJ202403 | C_AA107104.1 |

**Table S5. The information of all the 183 VZV strains in this study.**

| **SeqName** | **Lineage** | **Nation** | **Collect time** | **Vaccine** |
| --- | --- | --- | --- | --- |
| OQ718929.1 | Clade 1 | India | 2022/6/21 | no |
| NC001348.1 | Clade 1 (strain Dumas) | UK | 1986 | no |
| OQ916050.1 | Clade 5 | India | 2022/7/27 | no |
| KY037796.1 | Clade 2 | USA | 2012/3/19 | no |
| MH499466.1 | Clade 5 | India | Dec-17 | no |
| KY037798.1 | Clade 9 | USA | 2012/3/23 | no |
| KP702725.1 | Clade 6 | USA | 2013/3/27 | no |
| MH499468.1 | Clade 5 | India | Dec-17 | no |
| KY037797.1 | Clade 5 | USA | 2014/8/18 | no |
| KF811485.1 | Clade 2 | USA | 2012/4/8 | no |
| MH709349.1 | Clade 5 | USA | 2013/3/19 | no |
| MH709357.1 | Clade 4 | USA | 2013/4/14 | no |
| PP169944.1 | Clade 5 | Pakistan | Sep-23 | no |
| MH499467.1 | Clade 5 | India | Dec-17 | no |
| OQ427944.1 | Clade 5 | India | 2022/7/27 | no |
| MH709346.1 | Clade 3 | USA | 2013/2/27 | no |
| OQ835716.1 | Clade 9 | India | 2002/8/7 | no |
| MH709375.1 | Clade 3 | USA | 2013/7/10 | no |
| MT370830.1 | Clade 2 | South Korea Korea | 2012 | no |
| MH709341.1 | Clade 4 | USA | 2013/1/28 | no |
| MW545806.1 | Clade 2 | South Korea | 2017 | no |
| MT370828.1 | Clade 2 | South Korea | 2012 | no |
| MH709376.1 | Clade 2 | USA | 2013/6/21 | no |
| MT370829.1 | Clade 2 | South Korea | 2012 | no |
| MF898328.1 | Clade 2 | China | 2015 | Baike |
| OR148437.1 | Clade 5 | Pakistan | Apr-23 | no |
| MH709373.1 | Clade 5 | USA | 2013/6/20 | no |
| MH709359.1 | Clade 2 | USA | 2013/4/22 | no |
| MH709361.1 | Clade 1 | USA | 2013/4/29 | no |
| MH709362.1 | Clade 5 | USA | 2013/4/22 | no |
| MH709355.1 | Clade 2 | USA | 2013/3/2 | no |
| MH709360.1 | Clade 5 | USA | 2013/4/26 | no |
| MH709364.1 | Clade 4 | USA | 2013/5/8 | no |
| MH709377.1 | Clade 2 | USA | 2013/7/31 | no |
| OQ454913.1 | Clade 9 | India | 2022/8/13 | no |
| MT370826.1 | Clade 2 | South Korea | 2012 | VarilrixC |
| MH709363.1 | Clade 2 | USA | 2013/5/2 | no |
| OQ916049.1 | Clade 5 | India | 2022/7/17 | no |
| MH709336.1 | Clade 5 | USA | 2012/10/1 | no |
| MH709372.1 | Clade 4 | USA | 2013/6/27 | no |
| PP386307.1 | Clade 3 | Spain | 2007/6/18 | no |
| MH709353.1 | Clade 5 | USA | 2013/4/3 | no |
| MT370825.1 | Clade 2 | South Korea | 2013 | no |
| MH709339.1 | Clade 2 | USA | 2013/1/14 | no |
| MH709365.1 | Clade 5 | USA | 2013/5/12 | no |
| MH709374.1 | Clade 5 | USA | 2013/7/12 | no |
| MH709348.1 | Clade 2 | USA | 2013/3/18 | no |
| MW545807.1 | Clade 2 | South Korea | 2017 | no |
| MH709342.1 | Clade 5 | USA | 2013/2/10 | no |
| OQ723678.1 | Clade 5 | India | 2022/11/15 | no |
| MH709343.1 | Clade 5 | USA | 2013/2/8 | no |
| MH709371.1 | Clade 4 | USA | 2013/6/7 | no |
| MT370827.1 | Clade 2 | South Korea | 2012 | VarivaxD |
| MH709370.1 | Clade 4 | USA | 2013/6/1 | no |
| MH499469.1 | Clade 5 | India | Dec-17 | no |
| MH709351.1 | Clade 5 | USA | 2013/3/20 | no |
| MH709366.1 | Clade | USA | 2013/5/1 | no |
| MW545808.1 | Clade 2 | South Korea | 2017 | no |
| KU926314.1 | Clade 2 | South Korea | 2012 | VarivaxI |
| MH709350.1 | Clade 5 | USA | 2013/3/22 | no |
| MH709333.1 | Clade 2 | USA | 2012/9/27 | no |
| MH709345.1 | Clade 5 | USA | 2013/2/14 | no |
| MH709344.1 | Clade 2 | USA | 2013/2/9 | no |
| MH709334.1 | Clade 5 | USA | 2012/10/7 | no |
| MH709347.1 | Clade 5 | USA | 2013/3/13 | no |
| MH709340.1 | Clade 5 | USA | 2013/1/20 | no |
| MH709352.1 | Clade 9 | USA | 2013/4/3 | no |
| MH709335.1 | Clade 5 | USA | 2012/10/18 | no |
| DQ452050.1 | Clade 4 (M2DR) | USA | 2000 | no |
| MH709338.1 | Clade 5 | USA | 2012/10/1 | no |
| KU926322.1 | Clade 2 | South Korea | 2013 | no |
| KU926316.1 | Clade 2 | South Korea | 2012 | no |
| MH709331.1 | Clade 2 | USA | 2012/9/16 | no |
| DQ457052.1 | Clade 5 (CA123) | USA | 2006 | no |
| KU926312.1 | Clade 2 | South Korea | 2013 | no |
| KU926319.1 | Clade 2 | South Korea | 2012 | no |
| KU926313.1 | Clade 2 | South Korea | 2012 | VarilrixI |
| MH709337.1 | Clade 2 | USA | 2012/11/8 | no |
| MH709327.1 | Clade 5 | USA | 2012/8/14 | no |
| MH709328.1 | Clade 5 | USA | 2012/9/1 | no |
| KU926317.1 | Clade 2 | South Korea | 2012 | no |
| MH709309.1 | Clade 2 | USA | 2012/1/13 | no |
| MH709332.1 | Clade 5 | USA | 2012/9/20 | no |
| MH709318.1 | Clade 4 | USA | 2012/5/31 | no |
| KU926321.1 | Clade 2 | South Korea | 2012 | no |
| KU926311.1 | Clade 3 | South Korea | 2014 | no |
| MH709323.1 | Clade 4 | USA | 2012/6/25 | no |
| MH709329.1 | Clade 2 | USA | 2012/8/28 | no |
| DQ479955.1 | Clade 3 (11) | Canada | 2006 | no |
| MH709319.1 | Clade 2 | USA | 2012/6/2 | no |
| MH709322.1 | Clade 4 | USA | 2012/6/17 | no |
| DQ479953.1 | Clade 1 | Canada | 2006 | no |
| DQ479954.1 | Clade 1 | Canada | Apr-06 | no |
| MH709330.1 | Clade 5 | USA | 2012/9/10 | no |
| DQ479962.1 | Clade 1 | Canada | Apr-06 | no |
| KU926320.1 | Clade 2 | South Korea | 2012 | no |
| KC847290.1 | Clade 2 | China | 1984 | no |
| JN704694.1 | Clade 1 | Germany | 2007/11/7 | no |
| DQ479960.1 | Clade 4 (8) | Canada | 2006 | no |
| KU926315.1 | Clade 2 | South Korea | 2012 | no |
| DQ479961.1 | Clade 1 | Canada | Apr-06 | no |
| DQ008355.1 | Clade 2 | South Korea | 2012 | VariVax |
| MF004348.1 | Clade 2 | South Korea | 2012 | no |
| KJ767491.1 | Clade 2 | South Korea | 2012 | no |
| MH709324.1 | Clade 5 | USA | 2012/7/8 | no |
| MH709316.1 | Clade 4 | USA | 2012/5/16 | no |
| MH709326.1 | Clade 4 | USA | 2012/7/25 | no |
| MH709317.1 | Clade 3 | USA | 2012/5/30 | no |
| KC112914.1 | Clade 6 | Mexico | 2007 | no |
| KJ808816.1 | Clade 2 | South Korea | 2012 | no |
| JN704693.1 | Clade 1 | Germany | 2005/4/22 | no |
| JN704706.1 | Clade 5 | Germany | 2005/1/24 | no |
| JN704704.1 | Clade 5 | Germany | 2000/5/18 | no |
| MH709311.1 | Clade 2 | USA | 2012/3/10 | no |
| JQ972913.1 | Clade 3 | USA | 1964 | no |
| JN704707.1 | Clade 5 | Germany | 2007/3/15 | no |
| AJ871403.1 | Clade 3 | Germany | 2004 | no |
| KJ767492.1 | Clade 2 | South Korea | 2012 | no |
| MH709315.1 | Clade 2 | USA | 2012/5/13 | no |
| MH709310.1 | Clade 2 | USA | 2012/1/15 | no |
| AY548171.1 | Clade 1 | Canada | 2004 | no |
| MH709320.1 | Clade 2 | USA | 2012/6/9 | no |
| DQ674250.1 | Clade 1 | USA | Nov-00 | no |
| DQ479959.1 | Clade 1 | Canada | 2006 | no |
| AB097933.1 | Clade 2 (pOka) | Japan | 2002 | no |
| DQ479963.1 | Clade 1 | Canada | Apr-06 | no |
| MH709313.1 | Clade 2 | USA | 2012/3/18 | no |
| DQ479957.1 | Clade 3 (03-500) | Canada | 2006 | no |
| MH709314.1 | Clade 2 | USA | 2012/4/19 | no |
| JF306641.2 | Clade 2 | South Korea | 1989 | no |
| DQ008354.1 | Clade 2 | Belgium | 2005 | VarilRix |
| MH709325.1 | Clade 5 | USA | 2012/7/22 | no |
| JQ972914.1 | Clade 2 | USA | 1990 | no |
| JN704701.1 | Clade 3 | Germany | 2007/1/8 | no |
| JN704697.1 | Clade 2 | Germany | 2008/4/9 | no |
| C-TAN/BJ202306 | Clade 2 | China | 2023/7/16 | no |
| JN704700.1 | Clade 3 | Germany | 2005/1/3 | no |
| JN704699.1 | Clade 3 | Germany | 2003/11/22 | no |
| JN704696.1 | Clade 1 | Germany | 2005/4/22 | no |
| C-TAN/BJ202313 | Clade 2 | China | 2023/11/21 | no |
| JN704690.1 | Clade 1 | Germany | 2000/3/23 | no |
| PP261331.1 | Clade 2 | China | 2023/2/27 | no |
| DQ479958.1 | Clade 1 | Canada | 2006 | no |
| C-TAN/BJ202314 | Clade 2 | China | 2023/11/21 | no |
| C-TAN/BJ202307 | Clade 2 | China | 2023/7/24 | no |
| JN704705.1 | Clade 5 | Germany | 2004/5/7 | no |
| JN704703.1 | Clade 3 | Germany | 2007/7/26 | no |
| JN704698.1 | Clade 2 | Germany | 2008/4/9 | no |
| JN704710.1 | Clade 9 | Germany | 2008/2/8 | no |
| C-TAN/BJ202316 | Clade 2 | China | 2023/5/31 | no |
| EU154348.1 | Clade 1 | Russia | 1999 | no |
| MH709321.1 | Clade 5 | USA | 2012/6/14 | no |
| JN704708.1 | Clade 5 | Germany | 2007/10/30 | no |
| JN704702.1 | Clade 3 | Germany | 2007/3/8 | no |
| JN704691.1 | Clade 1 | Germany | 2004/7/8 | no |
| C-TAN/BJ202401 | Clade 2 | China | 2024/1/25 | no |
| MH709312.1 | Clade 9 | USA | 2012/4/15 | no |
| C-TAN/BJ202302 | Clade 2 | China | 2023/4/21 | no |
| JN704709.1 | Clade Ⅷ | Germany | 2005/9/17 | no |
| MH709358.1 | Clade 5 | USA | 2013/4/18 | no |
| AB097932.1 | Clade 2 | Japan | 2002/12/11 | vOka |
| C-TAN/BJ202310 | Clade 2 | China | 2023/12/3 | no |
| C-TAN/BJ202301 | Clade 2 | China | 2023/4/4 | no |
| C-TAN/BJ202309 | Clade 2 | China | 2023/11/22 | no |
| C-TAN/BJ202318 | Clade 2 | China | 2023/6/29 | no |
| AY548170.1 | Clade 1 (MSP) | Canada | Feb-04 | no |
| C-TAN/BJ202322 | Clade 2 | China | 2023/11/12 | no |
| C-TAN/BJ202305 | Clade 2 | China | 2023/6/29 | no |
| C-TAN/BJ202312 | Clade 2 | China | 2023/12/28 | no |
| C-TAN/BJ202324 | Clade 2 | China | 2023/12/24 | no |
| C-TAN/BJ202304 | Clade 2 | China | 2023/6/11 | no |
| C-TAN/BJ202311 | Clade 2 | China | 2023/12/17 | no |
| C-TAN/BJ202323 | Clade 2 | China | 2023/12/22 | no |
| C-TAN/BJ202308 | Clade 2 | China | 2023/11/17 | no |
| C-TAN/BJ202303 | Clade 2 | China | 2023/5/31 | no |
| C-TAN/BJ202402 | Clade 2 | China | 2024/2/21 | no |
| C-TAN/BJ202319 | Clade 2 | China | 2023/7/3 | no |
| DQ479956.1 | Clade 3 | Canada | 2006 | no |
| C-TAN/BJ202321 | Clade 2 | China | 2023/9/14 | no |
| C-TAN/BJ202403 | Clade 2 | China | 2024/1/6 | no |
| C-TAN/BJ202315 | Clade 2 | China | 2023/4/6 | no |
| JN704692.1 | Clade 1 | Germany | 2005/4/5 | no |
| JN704695.1 | Clade 1 | Germany | 2008/2/7 | no |

**Table S6. Positively Selected Sites (dN/dS >1) in VZV Genes.**

| **VZV protein (gene)** | **No. of positively selected residues** | **Aminoacid position(s)** |
| --- | --- | --- |
| envelope glycoprotein C (ORF14) | 20 | T37S, K39N, V44I, S51T, K53N, S65T, K67N, S79T, K81N, T93S, K95N, S107T, K109N, T121S, K123N, P124T, S135T, T144A, Y153F, A340T |
| tegument host shutoff protein (ORF17) | 4 | R140Q, T144A, T169M, V307I |
| capsid maturation protease (ORF33) | 3 | N313G, P374F, Y453S |
| capsid scaffold protein (ORF33.5) | 3 | N313G, P374F, Y453S |
| deoxyuridine triphosphatase (ORF8) | 0 | N/A |
| nuclear egress membrane protein (ORF24) | 0 | N/A |
| helicase-primase subunit (ORF52) | 0 | N/A |
| protein V57 (ORF57) | 0 | N/A |
| envelope glycoprotein L (ORF60) | 0 | N/A |

N/A-no data available.

**Table S7** **Positively selected sites in *ORF14* across 139 VZV strains.**

| **Site model (SM)** | | | | | | | | | |
| --- | --- | --- | --- | --- | --- | --- | --- | --- | --- |
| **Model** | **np** | **Ln L** | **Estimates of parameters** | | | | **Model compared** | **LRT P-value** | **Positive sites** |
| M3 | 281 | -2830.206004 | p: | 0.00000 | 0.99080 | 0.00920 | M0 vs. M3 | 0.000004927 | [] |
|  |  |  | ω: | 0.00000 | 0.21648 | 22.71093 |  |  |  |
| M0 | 277 | -2845.199019 | ω0: | 0.40855 | | |  |  | Not Allowed |
| M2a | 280 | -2830.205461 | p: | 0.99080 | 0.00000 | 0.00920 | M1a vs. M2a | 0.000184608 | [] |
|  |  |  | ω: | 0.21648 | 1.00000 | 22.71080 |  |  |  |
| M1a | 278 | -2838.802738 | p: | 0.74770 | 0.25230 |  |  |  | Not Allowed |
|  |  |  | ω: | 0.00000 | 1.00000 |  |  |  |  |
| M8 | 280 | -2832.145983 | p0=0.94482 | p=0.00500 | q=1.00784 |  | M7 vs.M8 | 0.000843966 | 6 D 0.613,158 T 0.642,286 E 0.564,291 H 0.591,313 N 0.999**,320 S 0.645,374 P 0.959*,432 D 0.543,434 R 0.622,453 Y 0.995**,478 H 0.591,488 W 0.676,514 T 0.581,528 H 0.645,559 N 0.553,561 T 0.580 |
|  |  |  | (p1= 0.05518) | ω= 7.70398 |  |  |  |  |  |
| M7 | 278 | -2839.223381 | p= | 0.00857 | q= | 0.01486 |  |  | Not Allowed |
| M8a | 279 | -2838.802855 | p0=0.74770 | p=0.00500 | q=1.59828 | | M8a vs.M8 | 0.000263468 | Not Allowed |
|  |  |  | (p1= 0.25230) | ω= 1.00000 | | |  |  |  |

**Table S8** **Positively selected sites in *ORF17* across 139 VZV strains.**

| **Site model (SM)** | | | | | | | | | |
| --- | --- | --- | --- | --- | --- | --- | --- | --- | --- |
| **Model** | **np** | **Ln L** | **Estimates of parameters** | | | | **Model compared** | **LRT P-value** | **Positive sites** |
| M3 | 281 | -2046.811777 | p: | 0.96282 | 0.03463 | 0.00255 | M0 vs. M3 | 0.000000000 | [] |
|  |  |  | ω: | 0.00000 | 15.75492 | 316.14692 |  |  |  |
| M0 | 277 | -2082.958433 | ω0: | 0.81549 | | |  |  | Not Allowed |
| M2a | 280 | -2053.164198 | p: | 0.83139 | 0.15414 | 0.01447 | M1a vs. M2a | 0.000000000 | [] |
|  |  |  | ω: | 0.00000 | 1.00000 | 51.45740 |  |  |  |
| M1a | 278 | -2075.810402 | p: | 0.77858 | 0.22142 |  |  |  | Not Allowed |
|  |  |  | ω: | 0.00000 | 1.00000 |  |  |  |  |
| M8 | 280 | -2054.021909 | p0=0.97485 | p=0.00761 | q=1.22144 |  | M7 vs.M8 | 0.000000000 | 129 N 0.685,140 R 1.000**,144 T 0.978*,160 D 0.681,168 K 0.695,169 T 0.971*,170 I 0.677,298 Q 0.716,307 V 0.972* |
|  |  |  | (p1= 0.02515) | ω= 38.20074 |  |  |  |  |  |
| M7 | 278 | -2076.357129 | p= | 0.00500 | q= | 0.00709 |  |  | Not Allowed |
| M8a | 279 | -2075.810812 | p0=0.77858 | p=0.00500 | q=1.44741 | | M8a vs.M8 | 0.000000000 | Not Allowed |
|  |  |  | (p1= 0.22142) | ω= 1.00000 | | |  |  |  |

**Table S9** **Positively selected sites in *ORF33* and *ORF33.5* across 139 VZV strains.**

| **Site model (SM)** | | | | | | | | | |
| --- | --- | --- | --- | --- | --- | --- | --- | --- | --- |
| **Model** | **np** | **Ln L** | **Estimates of parameters** | | | | **Model compared** | **LRT P-value** | **Positive sites** |
| M3 | 281 | -2830.206004 | p: | 0.00000 | 0.99080 | 0.00920 | M0 vs. M3 | 0.000004927 | [] |
|  |  |  | ω: | 0.00000 | 0.21648 | 22.71093 |  |  |  |
| M0 | 277 | -2845.199019 | ω0: | 0.40855 | | |  |  | Not Allowed |
| M2a | 280 | -2830.205461 | p: | 0.99080 | 0.00000 | 0.00920 | M1a vs. M2a | 0.000184608 | [] |
|  |  |  | ω: | 0.21648 | 1.00000 | 22.71080 |  |  |  |
| M1a | 278 | -2838.802738 | p: | 0.74770 | 0.25230 |  |  |  | Not Allowed |
|  |  |  | ω: | 0.00000 | 1.00000 |  |  |  |  |
| M8 | 280 | -2832.145983 | p0=0.94482 | p=0.00500 | q=1.00784 |  | M7 vs.M8 | 0.000843966 | 6 D 0.613,158 T 0.642,286 E 0.564,291 H 0.591,313 N 0.999**,320 S 0.645,374 P 0.959*,432 D 0.543,434 R 0.622,453 Y 0.995**,478 H 0.591,488 W 0.676,514 T 0.581,528 H 0.645,559 N 0.553,561 T 0.580 |
|  |  |  | (p1= 0.05518) | ω= 7.70398 |  |  |  |  |  |
| M7 | 278 | -2839.223381 | p= | 0.00857 | q= | 0.01486 |  |  | Not Allowed |
| M8a | 279 | -2838.802855 | p0=0.74770 | p=0.00500 | q=1.59828 | | M8a vs.M8 | 0.000263468 | Not Allowed |
|  |  |  | (p1= 0.25230) | ω= 1.00000 | | |  |  |  |

**Table S10. Statistical analysis of synonymous and nonsynonymous mutation frequencies in Beijing VZV strains.**

|  | **Tukey multiple comparison** | **Mean difference** | **Difference in 95.00% CI** | **Significant** | **Summary** | **Adjusted *P*-value** |
| --- | --- | --- | --- | --- | --- | --- |
| **N/S ratio** | Capsid/encapsidation vs. Envelope/glycoproteins | -0.4790 | -0.6046 to -0.3535 | yes | **** | <0.0001 |
|  | Capsid/encapsidation vs. Nuclear Egress | -0.1417 | -0.2478 to -0.03554 | yes | ** | 0.0046 |
|  | Capsid/encapsidation vs. Replication/regulatory | -0.003511 | -0.07798 to 0.07096 | no | ns | >0.9999 |
|  | Capsid/encapsidation vs. Tegument | -0.4918 | -0.6012 to -0.3823 | yes | **** | <0.0001 |
|  | Capsid/encapsidation vs. Unknown | 0.07860 | -0.06026 to 0.2175 | no | ns | 0.5143 |
|  | Envelope/glycoproteins vs. Nuclear Egress | 0.3374 | 0.1892 to 0.4855 | yes | **** | <0.0001 |
|  | Envelope/glycoproteins vs. Replication/regulatory | 0.4755 | 0.3565 to 0.5946 | yes | **** | <0.0001 |
|  | Envelope/glycoproteins vs. Tegument | -0.01274 | -0.1539 to 0.1285 | no | ns | 0.9997 |
|  | Envelope/glycoproteins vs. Unknown | 0.5576 | 0.3693 to 0.7460 | yes | **** | <0.0001 |
|  | Nuclear Egress vs. Replication/regulatory | 0.1382 | 0.01140 to 0.2649 | yes | * | 0.0271 |
|  | Nuclear Egress vs. Tegument | -0.3501 | -0.5014 to -0.1988 | yes | **** | <0.0001 |
|  | Nuclear Egress vs. Unknown | 0.2203 | 0.06720 to 0.3734 | yes | ** | 0.0021 |
|  | Replication/regulatory vs. Tegument | -0.4883 | -0.5555 to -0.4210 | yes | **** | <0.0001 |
|  | Replication/regulatory vs. Unknown | 0.08211 | -0.02293 to 0.1872 | no | ns | 0.1901 |
|  | Tegument vs. Unknown | 0.5704 | 0.4662 to 0.6746 | yes | **** | <0.0001 |
| **Nonsynonymous vs. synonymous** | N-Capsid/encapsidation vs. S-Capsid/encapsidation | 3.920 ± 0.2449 | 3.427 to 4.413 | yes | **** | <0.0001 |
|  | N-Envelope/glycoproteins vs. S-Envelope/glycoproteins | 0.3600 ± 0.5015 | -0.6483 to 1.368 | no | ns | 0.4763 |
|  | N-Nuclear Egress vs. S-Nuclear Egress | 1.400 ± 0.1633 | 1.072 to 1.728 | yes | **** | <0.0001 |
|  | N-Replication/regulatory vs. S-Replication/regulatory | 18.92 ± 0.5483 | 17.82 to 20.02 | yes | **** | <0.0001 |
|  | N-Tegument vs. S-Tegument | 0.5200 ± 0.6339 | -0.7546 to 1.795 | no | ns | 0.4161 |
|  | N-Unknown vs. S-Unknown | 1.960 ± 0.2688 | 1.419 to 2.501 | yes | **** | <0.0001 |

One-way ANOVA and t texts (GraphPad Prism 8). Significance thresholds: **P* ≤ 0.05, ***P* ≤ 0.01, ****P* ≤ 0.001, *****P* ≤ 0.0001 ns = not significant.
